# Insulin reverses impaired alveolar fluid clearance in ARDS by inhibiting LPS-induced autophagy and inflammatory

**DOI:** 10.3389/fimmu.2023.1162159

**Published:** 2023-08-15

**Authors:** Xu-peng Wen, Min Li, Ru-qi Zhang, Qi-quan Wan

**Affiliations:** ^1^ Transplantation Center, the Third Xiangya Hospital, Central South University, Changsha, Hunan, China; ^2^ Department of Critical Care Medicine, Zhongshan Hospital, Fudan University, Shanghai, China; ^3^ Department of Anatomy and Neurobiology, School of Basic Medical Sciences, Central South University, Changsha, Hunan, China

**Keywords:** ARDS, insulin, Na, K-ATPase, autophagy, inflammatory response

## Abstract

Until now, acute respiratory distress syndrome (ARDS) has been a difficult clinical condition with a high mortality and morbidity rate, and is characterized by a build-up of alveolar fluid and impaired clearance. The underlying mechanism is not yet fully understood and no effective medications available. Autophagy activation is associated with ARDS caused by different pathogenic factors. It represents a new direction of prevention and treatment of ARDS to restrain autophagy to a reasonable level through pharmacological and molecular genetic methods. Na, K-ATPase is the main gradient driver of pulmonary water clearance in ARDS and could be degraded by the autophagy-lysosome pathway to affect its abundance and enzyme activity. As a normal growth hormone in human body, insulin has been widely used in clinical for a long time. To investigate the association of insulin with Na, K-ATPase, autophagy and inflammatory markers in LPS-treated C57BL/6 mice by survival assessment, proteomic analysis, histologic examination, inflammatory cell counting, myeloperoxidase, TNF-α and IL-1β activity analysis etc. This was also verified on mouse alveolar epithelial type II (AT II) and A549 cells by transmission electron microscopy. We found that insulin restored the expression of Na, K-ATPase, inhibited the activation of autophagy and reduced the release of inflammatory factors caused by alveolar epithelial damage. The regulation mechanism of insulin on Na, K-ATPase by inhibiting autophagy function may provide new drug targets for the treatment of ARDS.

## 1 Introduction

Acute respiratory distress syndrome (ARDS) is a clinically life-threatening disease with poor prognosis and high treatment cost, characterized by intractable hypoxemia due to the accumulation of alveolar fluid. Severe pulmonary inflammation, damaged alveolar epithelium and impaired gas exchange with pulmonary edema are one of the main pathological features of ARDS (1, 2). The mechanisms of ARDS are intricately related to each other, which makes it extremely difficult to treat, and the mortality rate is as high as 35-55% (3, 4). For most patients with ARDS, effective removal of excess edema fluid from the alveoli and maintenance of a dry environment in the alveolar space are the main ways to relieve ARDS. In the past few years, researches on pulmonary water removal in ARDS have been in full swing, but no breakthroughs have been achieved so far.

Na, K-ATPase -mediated Na^+^ transport at the basolateral of alveolar type II epithelial (AT II) cells is the main driving force for alveolar fluid clearance (AFC) (5). The dysregulation of Na, K-ATPase in the ARDS state subsequently exacerbates pulmonary edema production mostly due to the restriction of Na^+^ transport and disruption of alveolar barrier function (6). Na, K-ATPase is a trimeric structure consisting of α, β and γ subunits. Among them, the α subunit plays a key role in edema fluid transporting (7). The α1, which carries several binding and functional domains, is not only most common in AT II cells and the main driver of intrapulmonary Na^+^ - K^+^ exchange but also facilitates AFC (8, 9). Intriguingly, Na, K-ATPase could be degraded via the autophagy-lysosome pathway, and it has been shown that the connection between the Na, K-ATPase and autophagy requires the involvement of the Na, K-ATPase α1 (10).

Pulmonary autophagy is a response of alveolar epithelial cells to long-term and sustained stimulation of external and internal factors. It maintains the balance of structure, metabolism and function of alveolar epithelial cells by phagocytosis of its cytoplasm or organelles and degradation in lysosomes (11). Several studies have shown that excessive autophagy exacerbates cellular injury. In contrast, inhibiting autophagy to a moderate level is beneficial in reducing lung injury in ARDS (12, 13). Studies have shown that Na, K-ATPase α1 and AMPK may serve as the “on” and “off” states of the autophagy pathway (10). Taken together, autophagy appears to contribute to endothelial barrier disruption caused by edema-inducing mediators, and inhibition of autophagy can protect against endothelial barrier function caused by ARDS.

Insulin has been reported to prevent or attenuate the occurrence of lipopolysaccharides (LPS)-induced acute lung injury in rats (14). Insulin upregulated the relative abundance of Na, K-ATPase on cell membranes and restored the efficiency of transporting (15). Therefore, insulin may act as a potential co-promoter of the Na, K-ATPase, suggesting that insulin may be promising as a therapeutic agent for ARDS. In our study, we found that LPS induced autophagy in ARDS mice, A549 cells and AT II cells, inhibited Na, K-ATPase α1 activity, and promoted the inflammatory response. Meanwhile, insulin inhibited their autophagy and inflammation levels and upregulated Na, K-ATPase function. Our study reveals that insulin is likely to regulate Na, K-ATPase α1 expression by inhibiting autophagy and thus reversing the impaired AFC in ARDS caused by LPS, and the effect of insulin on inflammatory response may also improve the prognosis of ARDS by targeting various key factors which promote AFC.

## 2 Materials and methods

### 2.1 Animal experiment

All experimental animal treatment procedures were approved by the Department of Laboratory Animals of Xiangya School of Medicine, Central South University, and are carried out under the Guide for the Use of Laboratory Animals by the National Institutes of Health. The male C57BL/6 mice from 6 to 8 weeks old were purchased from the Department of Laboratory Animals of Xiangya School of Medicine, Central South University (Changsha, China). All mice were housed in specific pathogen-free conditions at 22°C environment under 12 h light/dark cycle.

### 2.2 *In vivo* model of ARDS

A total of 120 mice were randomly divided into different groups according to different experimental requirements. To assess mortality, mice are treated with different doses (0.5, 1.0 and 1.5 IU/kg) of insulin 0.5 h after injection of 10 mg/kg LPS intratracheally. Different doses of insulin were dissolved in different concentrations of glucose solution (1%, 5%, 10%) to avoid hypoglycemia and to keep the blood glucose concentration in the appropriate range. The mortality of the mice was recorded every 12 h for 3 days after LPS injection. The experiments were performed using mice from the same litter, with 10 mice in each group.

To further investigate the effect of insulin on ARDS, mice were randomly divided into four groups: 1) control group (n=6): mice received normal saline (NS) instilled intratracheally; 2) LPS group (n=6): mice were administered LPS (10 mg/kg) instilled intratracheally; 3) Insulin group (n=6): mice were given insulin(1.0 IU/kg) under subcervical skin 0.5 h after saline administration (n=6); 4) LPS+insulin group(n=6) mice received insulin (1.0 IU/kg) 0.5 h after LPS (10 mg/kg) administration (n=6).

**Figure 1:**
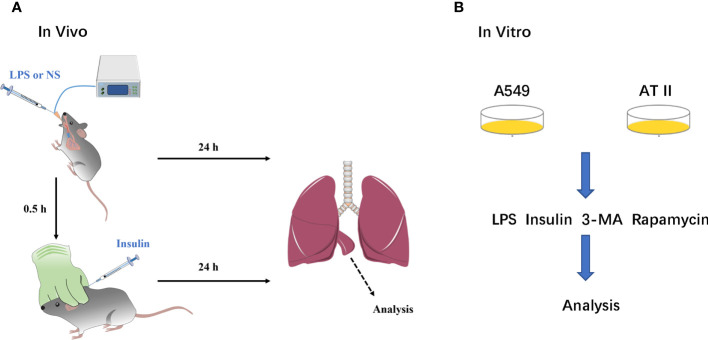
A simple flow chart of animals and cells experiments. **(A)** ARDS animals model and insulin intervention; **(B)** ARDS cells model and insulin intervention.

Mice were anesthetized by using sodium pentobarbital (30 mg/kg, i.p.) throughout modeling. LPS and saline were administered via a tracheal tube connected to the animal’s ventilator, which allows the LPS and saline to be evenly distributed in the lungs of the mice. Insulin and saline were administered by subcutaneous injection under the neck. A blood glucose meter detected blood glucose level during drug administration (**Figure 1**).

### 2.3 Pulmonary histopathology

The lung tissues were fixed in 4% formaldehyde, embedded in paraffin and cut into 5 μm thick sections. Next, the sections were stained with hematoxylin-eosin (HE). Changes in lung histopathology were observed under the microscope and a pathology score was obtained. Depending on the degree of lung injury, hemorrhage, edema, exudation, necrosis, congestion, neutrophil infiltration, and pulmonary atelectasis, the score is based on a scale of 0 to 4 (16): no injury = 0, lesion field <25% = 1, lesion field 25-50% = 2, lesion field 50-70% = 3, full field of vision = 4. Scores were calculated for statistical analysis.

### 2.4 Inflammatory cell counting and protein concentration determination in the BALF

The collected bronchoalveolar lavage fluid (BALF) was centrifuged at 1000×g for 15 minutes at 4°C, the supernatant was collected and frozen at -80°C for subsequent assays. The total number of inflammatory cells in the BALF was determined by counting cells with a blood cell counter (Beckman Coulter, Inc.) after resuspension of the cell precipitate in PBS and exclusion of dead cells by Tissue Blue staining. For analysis of cell numbers, 100 μl of BALF was centrifuged onto slides by Cytospin (Thermo Fisher Scientific, Waltham, USA). After the slides were dried, the cells were fixed and stained using Wright’s staining solution (32857, Sigma, USA) according to the manufacturer’s instructions. The number of neutrophils was sorted to determine the percentage of neutrophils by a laboratory technician who was unaware of the experimental design. The frozen BALF supernatant was thawed and mixed thoroughly and the total protein concentration was determined by the BCA (bicinchoninic acid) method.

### 2.5 Lung wet/dry ratios

Mice were sacrificed and the lung was removed and aspirated. Wet weights were obtained immediately. The lungs were then dried in an oven at 80°C for 48 hours to obtain a dry weight. Lung tissue edema was assessed by calculating the ratio of wet to dry lungs.

### 2.6 Enzyme-linked immunosorbent assay

Collect BALF as described above. Myeloperoxidase (MPO) was measured in BALF using MPO ELISA kits (Cusabio, China) per the manufacturer’s instructions. And the levels of TNF-α and IL-1β in serum were determined using ELISA kits according to the manufacturer’s instructions.

### 2.7 Cell culture and treatment

We performed the isolation, culture and purification of mouse primary AT II cells: According to the Elise M method (17).A549 cell line was purchased from ATCC. AT II and A549 cells were cultured in DMEM (Gibco, USA) supplemented with 10% FBS (Gibco, USA) and 1% penicillin/streptomycin (Cytiva, USA). LPS was administered to AT II and A549 cells at 1μg/ml for 12h and insulin (100nM) was administered at the eighth hour (**Figure 1**).

### 2.8 Cell viability assay

Cell viability was assessed by using the CCK-8 kit (APE Bio, USA). CCK-8 solution (20 μl) was added to 200 μl of a complete medium in each well of the 96 wells and incubated for 30 minutes. The absorbance value at 450 nm was measured using a microplate reader.

### 2.9 Transmission electron microscopy

A549 cells were collected, washed 3 times with 0.1 M PBS and prefixed with a 3% glutaraldehyde, then postfixed in 1% osmium tetroxide, dehydrated in series acetone, infiltrated in Epox 812 for a longer, and embedded, fixed overnight at 4°C in 2.5% glutaraldehyde, then dehydrated using an acetone gradient, embedded, sectioned and routinely stained. Semi-thin sections were stained with methylene blue, ultra-thin sections were cut with a diamond knife and stained with uranyl acetate and lead citrate sections were examined with HT7800 transmission electron microscope from HITACHI to visualize autophagosomes and autolysosomes.

### 2.10 Proteomic analysis

We used the label-free quantification for proteomic analysis, with each sample prepared and detected by LC-MS/MS independently, as described in the **Supplementary Material**. Data are available via ProteomeXchange with identifier PXD040288.

### 2.11 Western blot

Protein preparation and western blot detection as previously described (18). The lung tissues, A549 cells and AT II cells were lysed with RIPA lysis buffer (Beyotime, China) on ice for 1h. Then, electrophoresed on 10-12% SDS-PAGE gels before being transferred to PVDF membranes (millipore). After blocking with 5% nonfat dry milk for 1 h, the membrane was left overnight at 4°C with the primary antibody against Beclin1, light chain 3 (LC3), P62/SQSTM1, ATG5, Na, K-ATPase α1(ATP1A1), Na, K-ATPase β1(ATP1B1), GAPDH and β-actin followed by 2 h incubation with an appropriate peroxidase-conjugated rabbit or mouse secondary antibody. The immune-reactive bands were visualized by enhanced chemiluminescence and exposed to the gel imaging system. Immunoreactive bands were analysed using image analysis software with the ECL system. The gel images were analyzed with the Image J software.

### 2.12 Statistical analysis

Distributed data are presented as means ± standard deviation (SD). Experimental data were compared between two independent groups by unpaired Student’s t-test. The difference across multiple groups was assessed by one-way analysis of variance (ANOVA), followed by a Tukey test for multiple comparisons. Survival data was presented by the Kaplane-Meier method and comparisons were made by the log rank test All data processing using the GraphPad 8.0 software. Statistical significance was established at *P* < 0.05.

## 3 Results

### 3.1 Insulin reduces cumulative mortality in LPS-induced ARDS mice

**Figure 2:**
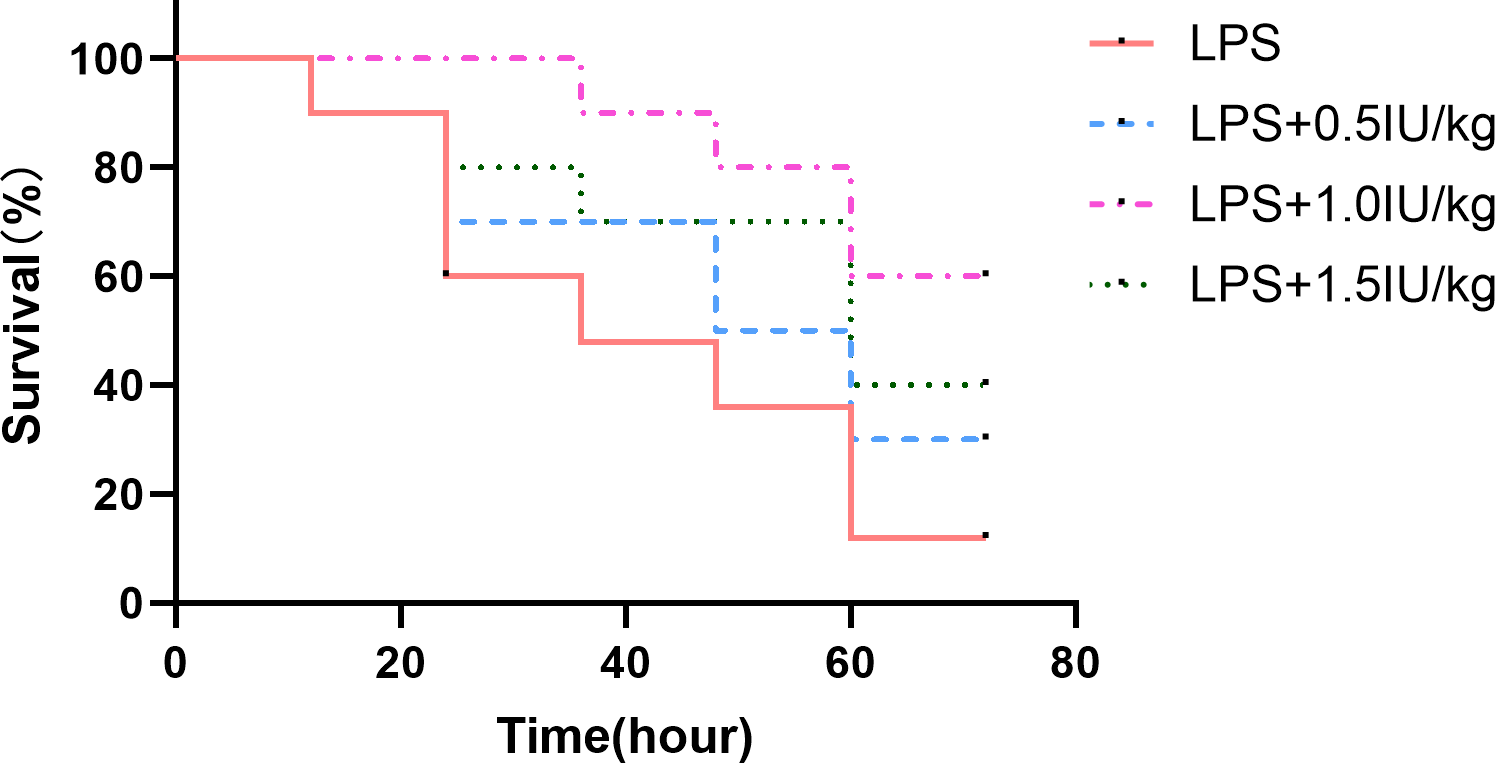
Effect of different concentrations of insulin on survival in ARDS mice. Mice were challenged with LPS (10 mg/kg) with or without different doses of insulin treatment (0.5, 1.0 and 1.5 IU/kg). Survival was observed for 12, 24, 36, 48, 60 and 72 h. Experiments were performed using mice from the same litter, each group contains 10 mice.

To verify whether insulin has a therapeutic effect on the ARDS model and determine the concentration of insulin to be used in subsequent experiments, we used insulin (0.5/1.0/1.5 IU/kg) which were administered 0.5 h after LPS (10 mg/kg) administration, and found that (as shown in **Figure 2**) insulin significantly improved the survival of LPS-induced ARDS mice, with cumulative survival rates of 60% and 40% during 3 days in the 1.0 IU/kg and 1.5 IU/kg insulin groups, respectively, significantly higher than that in the LPS group (10%, *P* < 0.01), and the cumulative survival rate was higher at 1.0 IU/kg (60%) than at 1.5 IU/kg (40%) (*P* < 0.05) insulin group. No preventive effect of 0.5 IU/kg insulin on death (30%, *P* > 0.05). Therefore, we chose 1.0 IU/kg insulin to go for the subsequent series of experiments.

### 3.2 Proteomics found that autophagy-lysosome pathway plays an indispensable role in the treatment of ARDS mice with insulin

**Figure 3:**
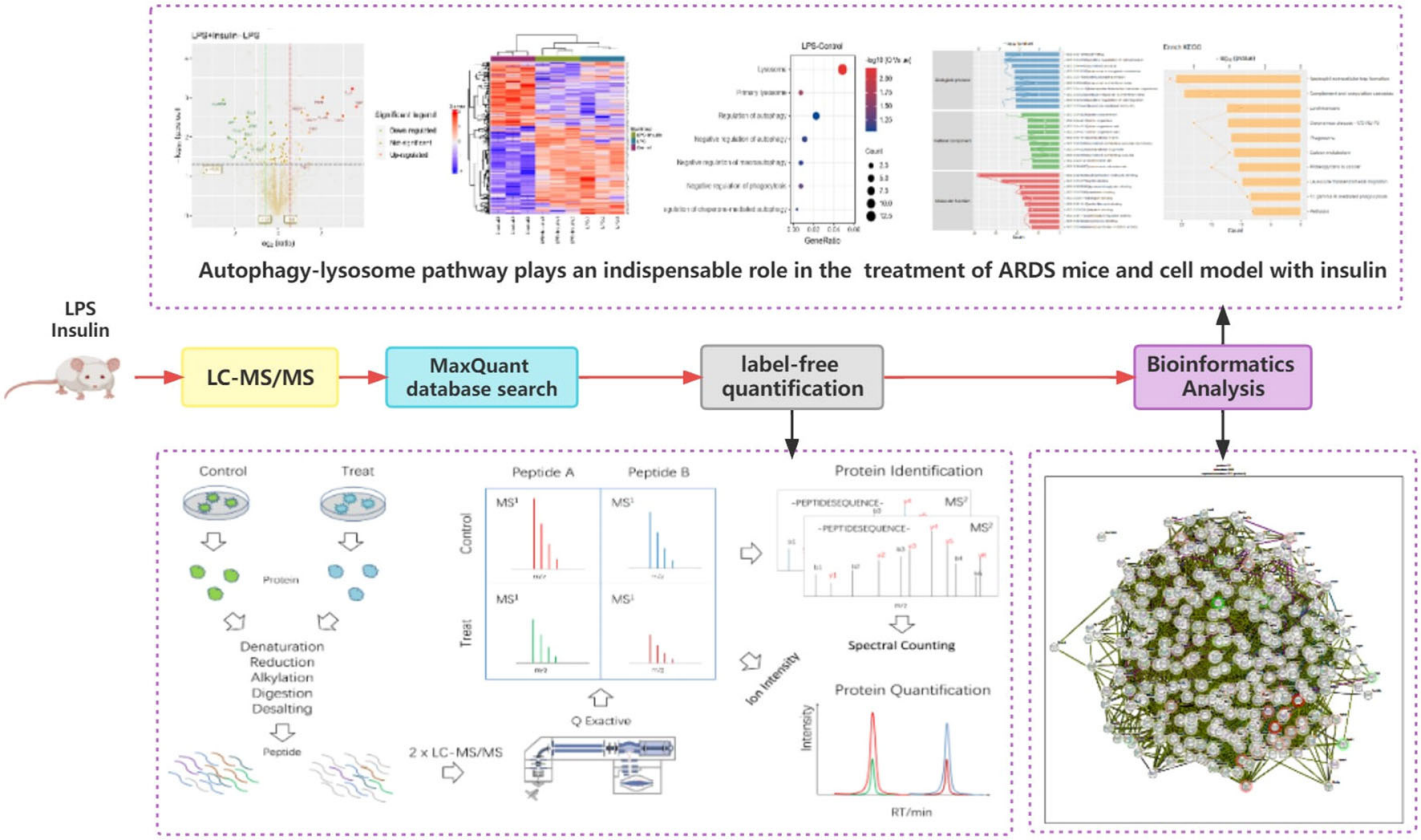
Proteomics Graphical Abstracts.

**Figure 4:**
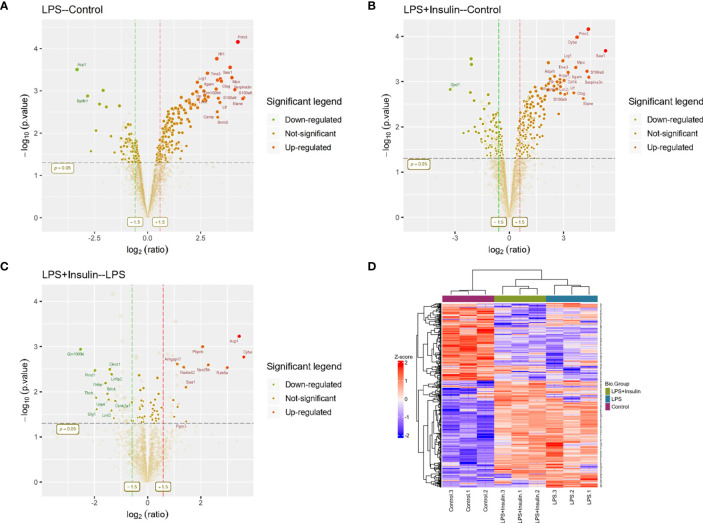
Linkage of insulin and LPS-induced ARDS by proteomics. **(A–C)** Volcano plots for LPS-Control, LPS+Insulin-Control and LPS+Insulin-LPS. Proteins with significant differences between the two groups are indicated in dark green (down-regulated) and orange-red (up-regulated), respectively. Proteins with no statistical difference are shown in brown. **(D)** Differential protein hierarchical clustering analysis, the top color bar represents the grouping of samples; the bottom is the corresponding sample name; the protein name on the right corresponds to the symbol column in the differential protein list (Supplemental Material.csv).

To understand the specific regulatory mechanism of insulin in the process of ARDS and reveal the differential expression of related proteins after ARDS, we established the interaction group of insulin and LPS at the protein expression level by label-free quantification (**Figure 3**). C57 mice were treated with LPS to simulate inflammatory injury and additionally intervened with insulin. For group comparisons, we used volcano plots to display (**Figures 4A–C**) up- and down-regulated proteins for each group. To test the rationality and accuracy of the selected differentially expressed proteins, we used the screened proteins to perform hierarchical clustering on each group of samples. Compared with the control group (Control), the interacting proteins of the experimental group (LPS, LPS + Insulin) were significantly enriched, which indicates that the differentially expressed proteins have higher specificity (**Figure 4D**). Notably, we ranked the proteins in the LPS and LPS+Insulin groups according to Fold Change (FC) values and listed the top 10 candidate proteins that were significantly up- or down-regulated as following **Table 1**.

**Table 1:** Up- or down-regulated proteins in the top 10 FC rankings in LPS+Insulin group vs LPS group.

Rank	LPS-A549 vs Control-A549		
	Annotation	Alias	FC*
1	keratin 17	KRT17	3.20673671
2	dihydrolipoamide dehydrogenase	DLD	2.619500792
3	nicalin	NCLN	2.355100614
4	mutS homolog 2	MSH2	2.355100614
5	heterogeneous nuclear ribonucleoprotein H3 (2H9)	HNRNPH3	1.619500792
6	stearoyl-CoA desaturase (delta-9-desaturase)	SCD	1.619500792
7	LIM and SH3 protein 1	LASP1	1.451281189
8	coatomer protein complex, subunit beta 2 (beta prime)	COPB2	1.451281189
9	coiled-coil domain containing 86	CCDC86	1.355100614
10	solute carrier family 1 (neutral amino acid transporter), member 5	SLC1A5	1.355100614
Down-regulated Proteins			
1	ribosomal RNA processing 1 homolog B	RRP1B	-3.459431619
2	dolichyl-phosphate mannosyltransferase polypeptide 1	DPM1	-2.584962501
3	GDP dissociation inhibitor 2	GDI2	-2.321928095
4	nucleoporin 107kDa	NUP107	-2.321928095
5	talin 1	TLN1	-2.321928095
6	WD repeat domain 36	WDR36	-2.321928095
7	WD repeat domain 46	WDR46	-2.149102965
8	DEAH (Asp-Glu-Ala-Asp/His) box polypeptide 57	DHX57	-2.000000000
9	G1 to S phase transition 2	GSPT2	-2.000000000
10	nuclear factor of kappa light polypeptide gene enhancer in B-cells 2	NFKB2	-2.000000000

**Figure 5:**
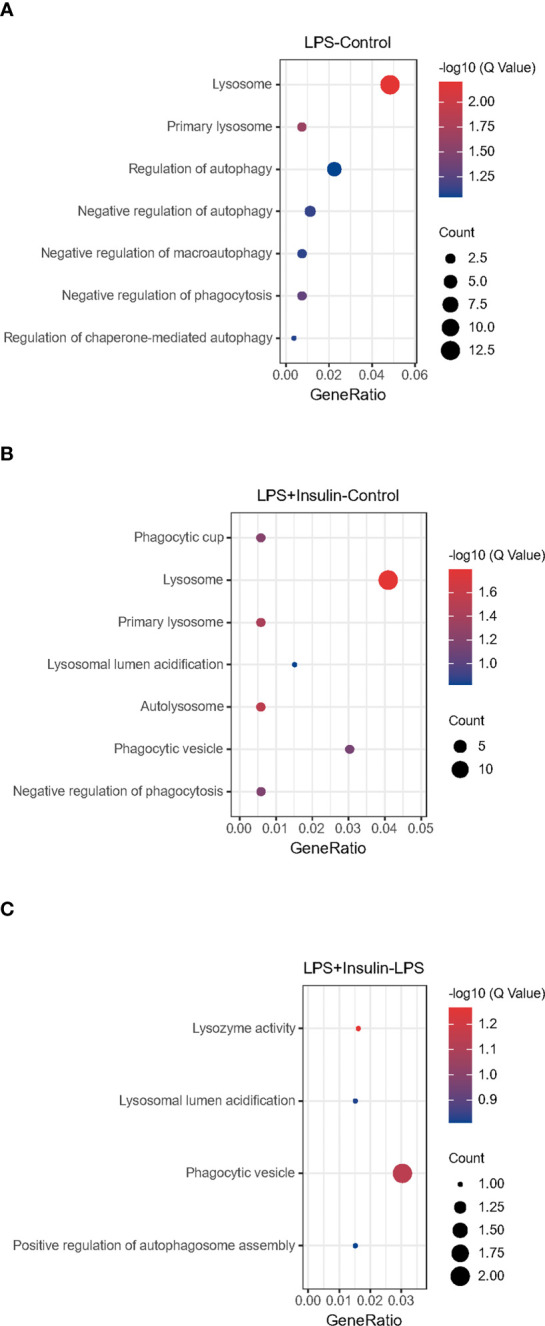
Gene Ontology analysis **(A–C)** Gene Ontology analysis revealing enrichment of biological process terms of differentially expressed proteins under LPS-Control, LPS+Insulin-Control and LPS+Insulin-LPS.

After that, we tried to use GO enrichment analysis to study the biological processes that differentially expressed proteins participated in. Interestingly, we found that many proteins are all related to autophagy-lysosomal degradation in the normal group (Control), ARDS group (LPS), and treatment group (LPS + Insulin) (**Figure 5**). These MS-based GO analysis data provide us with a clue that insulin may regulate the occurrence and development of ARDS through the autophagy-lysosomal degradation system.

### 3.3 Insulin alleviates symptoms of LPS-induced ARDS mice and exerts anti-inflammatory effect.

**Figure 6:**
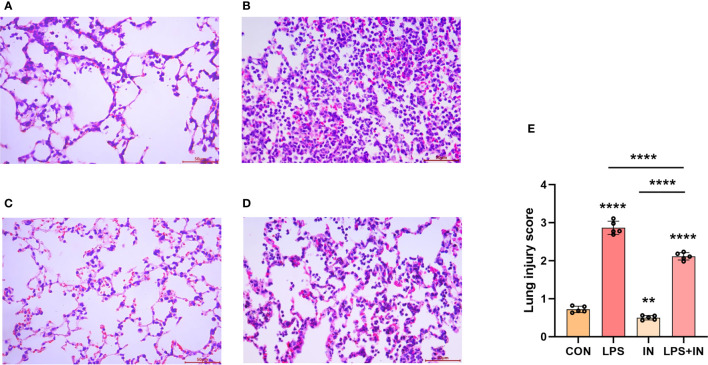
Effects of insulin on lung histopathological changes in LPS-induced ARDS mice. Mice were challenged by LPS (10 mg/kg) with or without insulin treatment (1.0 IU/kg). Representative histological changes of lung obtained from mice of different groups. **(A)** Control group, **(B)** LPS group, **(C)** Insulin group, **(D)** LPS + insulin group, (Hematoxylin and eosin staining, Scale bar, 50μm). **(E)** Lung histologic injury score. The data are presented as mean ± SD. n = 5, the horizontal line represents the comparison between each two groups, **p < 0.01.****p < 0.0001.

To explain whether insulin alleviated inflammation of ARDS induced by LPS *in vivo*, we developed the ARDS mouse model induced by LPS administration, and evaluated the changes of pulmonary histopathological features by HE staining, lung W/D ratio and lung injury score. It was indicated that the lung tissues accompanied with inflammatory cell infiltration, edema, vascular congestion and alveolar wall thickening in ARDS model induced by LPS (**Figures 6A–D**). However, insulin significantly attenuated LPS-induced histopathological changes. Importantly, a scoring system was used to assess the degree of lung injury. As shown in (**Figure 6E**), the quantitative scoring of histological lung injury in the ARDS mice was markedly increased compared with that in the control group 24 h after LPS challenge. However, insulin markedly decreased the pathological scores compared with those in the LPS group.

**Figure 7:**
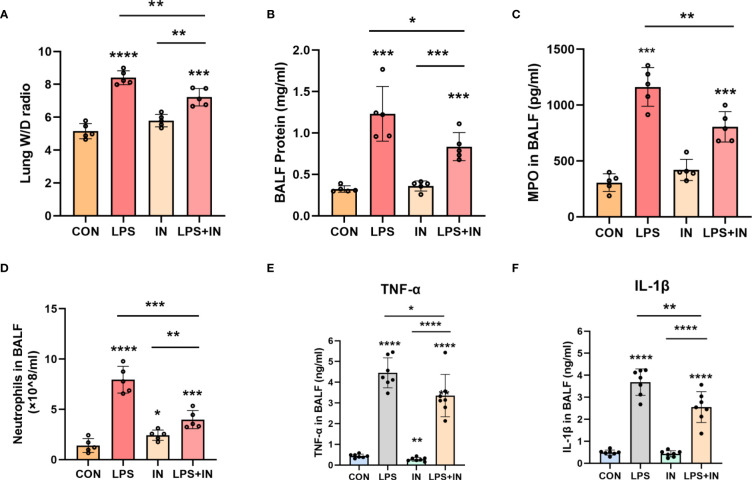
Effects of insulin on pulmonary edema and inflammation in LPS-induced ARDS mice. Treat the mice as described in Materials and Methods. **(A)** Lung W/D ratio. **(B)** BALF protein concentration. **(C)** MPO in BALF. **(D)** Neutrophils in BALF. **(E, F)** Effects of insulin on TNF-α and IL-1β production in the BALF. The data are presented as mean ± SD. n = 5, the horizontal line represents the comparison between each two groups, *p<0.05. **p < 0.01. ***p < 0.001. ****p < 0.0001.

Lung W/D ratio and BALF total protein concentration, two commonly used indicators of pulmonary vascular permeability, are important features of ARDS. Importantly, the lung W/D ratio and BALF total protein concentration dramatically increased after LPS administration, and the phenomena were reversed with insulin treatment (*P* < 0.05) (**Figures 7A, B**). Meanwhile, compared with those in the LPS group, insulin significantly inhibited the increase of MPO activity and the number of neutrophil cells induced by LPS (**Figures 7C, D**). Besides, to investigate the anti-inflammatory effect of insulin, the levels of TNF-α and IL-1β in BALF were detected in this study. As shown in **Figures 7E, F**, compared with the normal control group, the levels of TNF-α and IL-1β in the BALF of the LPS group were significantly increased. Compared with the LPS group, the levels of TNF-α and IL-1β in the BALF of the LPS+insulin group were significantly decreased. These results suggest that insulin attenuates pulmonary edema and inflammation in LPS-challenged mice.

### 3.4 Insulin regulates the expression of the Na, K-ATPase by inhibiting autophagy in LPS-induced ARDS mouse model

**Figure 8:**
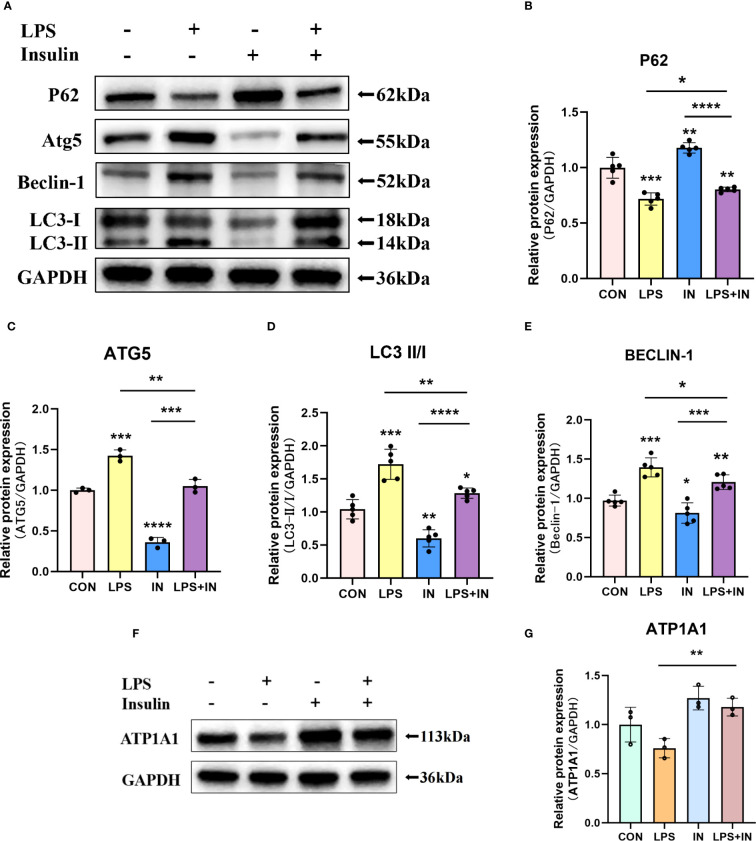
Effects of insulin on autophagy-related proteins and ATP1A1 expression in LPS-induced ARDS mice. Treat the mice as described in Materials and Methods. **(A, F)** Representative western blotting detected the levels of LC3-II/I, Beclin-1, ATG5, P62 and ATP1A1 in lung tissues **(B–E, G)** Quantitative analysis of LC3-II/I, Beclin-1, ATG5, P62 and ATP1A1 were shown in bar graphs, respectively. The data are presented as mean ± SD. n = 3, the horizontal line represented the comparison between each two groups, *p<0.05. **p < 0.01. ***p < 0.001. ****p < 0.0001.

Na, K-ATPase participates in the autophagy-lysosome pathway through its α1 subunit, and ATP1A1 may act as a shutdown factor of the autophagy pathway (10). To explain whether insulin reduces LPS-induced ARDS inflammation and improves Na, K-ATPase by inhibiting autophagy *in vivo*, we examined autophagy levels in an LPS-induced ARDS mouse model. We first assessed the protein levels of LC3-II/I, Beclin-1, ATG5 and P62 by western blotting. As shown in **Figures 8A–E**, LC3-II/I, Beclin-1 and ATG5 expression were significantly elevated and P62 expression was decreased in the LPS group. In particular, the treatment of insulin resulted in remarkably decreased LC3-II/I, Beclin-1 and ATG5 expression, while p62 accumulation was effectively enhanced in the LPS-induced ARDS mouse model. Our results suggest that LPS-induced autophagy activation was reversed by insulin *in vivo*. Furthermore, the reduce in ATP1A1 accumulation following exposure to LPS was attenuated considerably by insulin (**Figures 8F, G**). It can be seen that inhibiting autophagy may be crucial for regulating the expression of Na, K-ATPase in the lung tissue, improving the inflammatory response, and inhibiting the generation of pulmonary edema fluid.

### 3.5 Insulin attenuates autophagy levels and improves Na, K-ATPase expression in LPS-induced A549 cells

**Figure 9:**
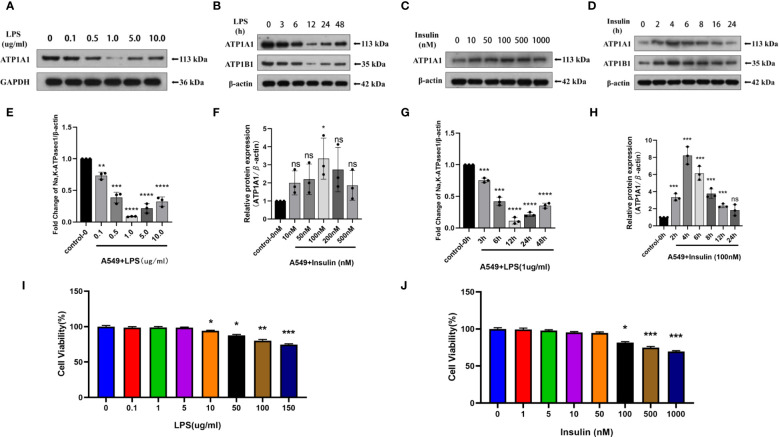
Effects of LPS and insulin on Na, K-ATPase expression and cellular activity in A549 cells. **(A, B, E, F)** Representative western blotting detected the levels of ATP1A1 in A549 cells by different LPS and insulin concentrations and duration **(C, D, G, H)** Quantitative analysis of ATP1A1 was shown in bar graphs, respectively. **(I, J)** Effects of different doses of LPS and insulin on the survival rate of A549 cells using CCK-8 assay. The data are presented as mean ± SD. n = 3, the horizontal line represented the comparison between each two groups, *p<0.05. **p < 0.01. ***p < 0.001. ****p < 0.0001.

To directly investigate the role of LPS and insulin in the development of ARDS *in vitro*, we utilized LPS-induced human AT II cell line (A549) as a model of ARDS. To determine the optimal concentration and duration of action of LPS and insulin affecting ATP1A1 expression in A549 cells, we first utilized western blotting to detect ATP1A1 expression levels, and the results showed that ATP1A1 was significantly inhibited at LPS concentration of 1.0μg/ml after using separately LPS at 0μg/ml, 0.1μg/ml, 0.5μg/ml, 1.0μg/ml, 5.0μg/ml, and 10.0μg/ml(**Figures 9A, C**). Subsequently, after using 1.0μg/ml to act on A549 cells for 0h, 3h, 6h, 12h, 24h, and 48 h, respectively, it was found that 12 h was the suitable time period when ATP1A1 was significantly inhibited (**Figures 9B, D**). Similarly, we puzzled over a range of insulin concentrations and times. We found that the expression of ATP1A1 increased significantly when the insulin concentration was 100 nM and acted for 4h (**Figures 9E–H**).

To ensure the accuracy and stability of subsequent experiments, we also examined the effects of concentration gradients of LPS and insulin on cell viability using CCK-8 assay. Which showed that after 12h and 4h of LPS and insulin acting on A549 cells, respectively the viability of A549 cells decreased with increasing doses of LPS and insulin in a concentration-dependent manner (**Figures 9I, J**). Combining the above data, we chose 1 μg/ml LPS and 100 nM insulin to act on A549 cells for 12h and 4h, respectively, for subsequent cell-modeling doses of ARDS.

**Figure 10:**
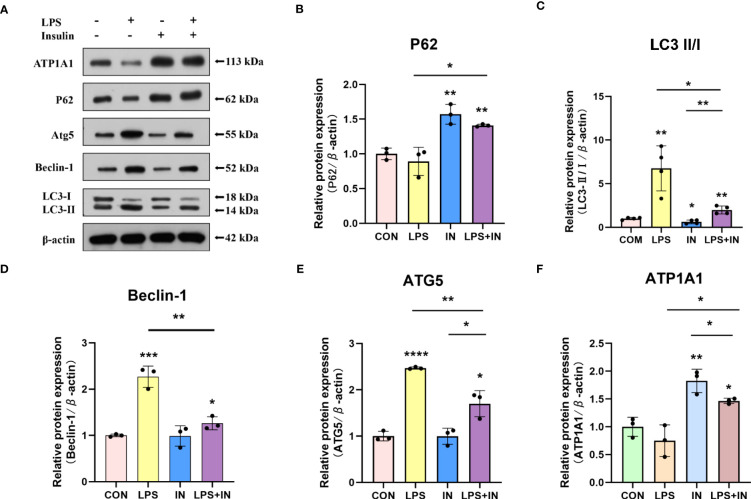
Effects of insulin on autophagy-related proteins and ATP1A1 expression in LPS-induced A549 cells. **(A)** Representative western blotting detected the levels of LC3-II/I, ATG5, Beclin-1, P62 and ATP1A1 **(B–F)** Quantitative analysis of LC3-II/I, Beclin-1, ATG5, P62 and ATP1A1 were shown in bar graphs, respectively. The data are presented as mean ± SD. n = 3, the horizontal line represented the comparison between each two groups, *p<0.05. **p < 0.01. ***P < 0.001. ****P < 0.0001.

Intriguingly, to investigate whether the protective effect of insulin on LPS-induced Na, K-ATPase in A549 cells is related to autophagy, we detected the levels of several key autophagy-related proteins using western blotting. A549 cells were treated with LPS for 12 hours, and insulin was administrated at the eighth hour. As shown in the **Figures 10A–E**, LPS treatment significantly up-regulated LC3-II/LC3-I, ATG5 and Beclin-1 levels, while P62 decreased, suggesting autophagy activation (*P* < 0.01). On the contrary, insulin can reverse the activation of autophagy and play an inhibitory role (*P* < 0.05). Subsequently, insulin treatment significantly up-regulated the expression of ATP1A1, which was inhibited by LPS (**Figure 10F**), which indicated that insulin increased the expression of Na, K-ATPase in LPS-treated cells, which may be related to the inhibition of autophagy. The above results indicate that insulin inhibited autophagy and improved Na, K-ATPase expression in ARDS *in vitro*, which may play an important role in limiting pulmonary edema.

### 3.6 Insulin improves Na, K-ATPase expression by inhibiting autophagy in ARDS which was confirmed by autophagy inhibitors and promoters

To make our results more robust and accurate, we administered the autophagy inhibitor 3-methyladenine (3-MA, 3mM) and the autophagy promoter rapamycin (1nM) 30min before LPS induction and then intervened with insulin in A549 cells after LPS administration.

**Figure 11:**
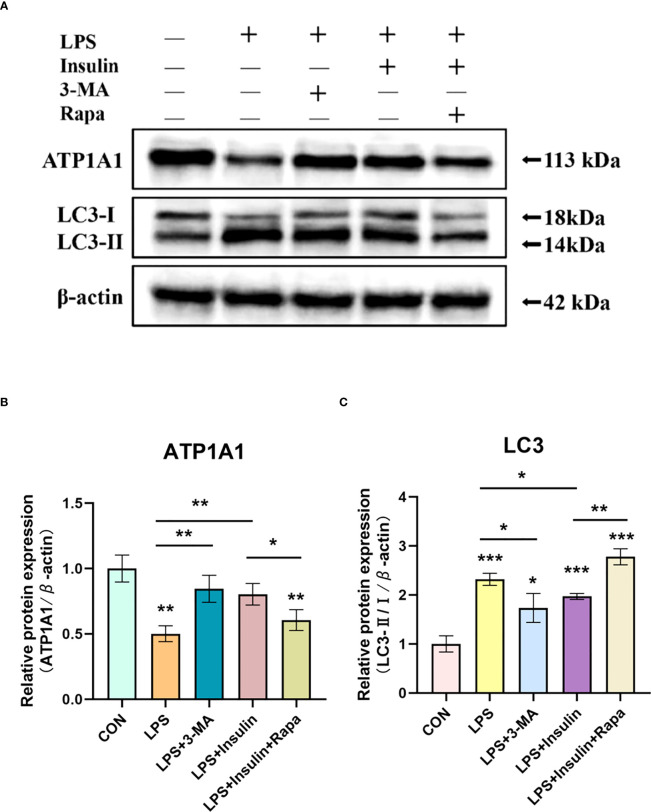
Effect of 3-MA and Rapamycin on the role of insulin in LPS-induced autophagy in A549 cells. **(A) **Western blot assay for LC3-II/I and ATP1A1. **(B, C)** Quantitative analysis. The data are presented as mean ± SD. n = 3, the horizontal line represented the comparison between each two groups, *P < 0.05, **P < 0.01, ***P < 0.001.

Notably, we obtained better results. It was found that in the LPS+3-MA group compared with the LPS group, the expression level of ATP1A1 increased, suggesting that inhibition of autophagy could restore the level of ATP1A1 inhibited by LPS to some extent. And the same effect was exerted in the LPS+insulin group. In addition, the administration of Rapamycin on top of LPS and insulin revealed that the expression level of ATP1A1 decreased, suggesting that the effect of insulin could be attenuated after promoting autophagy (**Figures 11A–C**). The above results further demonstrate that insulin inhibits autophagy and improves the expression of Na, K-ATPase in ARDS *in vitro*, which may play an important role in limiting pulmonary edema.

### 3.7 Ultrastructure of A549 cells induced by LPS after insulin administration observed by TEM

**Figure 12:**
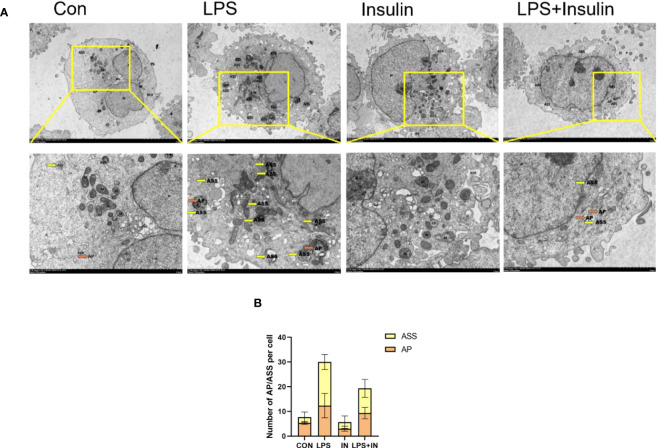
The remarkable images of autophagosomes ultrastructure by TEM in A549 cells **(A)** Representative photographs of TEM showed autophagosomes and autolysosomes. **(B)** Quantitative analysis of autophagosomes (orange dots) and autolysosomes (yellow dots), bars represented as mean ± SD.

Using transmission electron microscopy (TEM) to observe cell autophagy and its morphological structure is currently a highly recommended and more accurate method for autophagy detection. To further study the effect of insulin on the number of autophagosomes (AP) and autolysosomes (ASS) induced by LPS in A549 cells, we used TEM to observe them compared with the control group. It revealed that the number of AP and ASS increased after LPS treatment, and decreased in the insulin group. Compared with the LPS group, The number of AP and ASS decreased significantly in the LPS+insulin group (**Figures 12A, B**). Therefore, insulin may inhibit the autophagy activated by LPS in cells.

### 3.8 Insulin attenuates autophagy levels and improves Na, K-ATPase expression in LPS-induced mouse primary AT II cells

**Figure 13:**
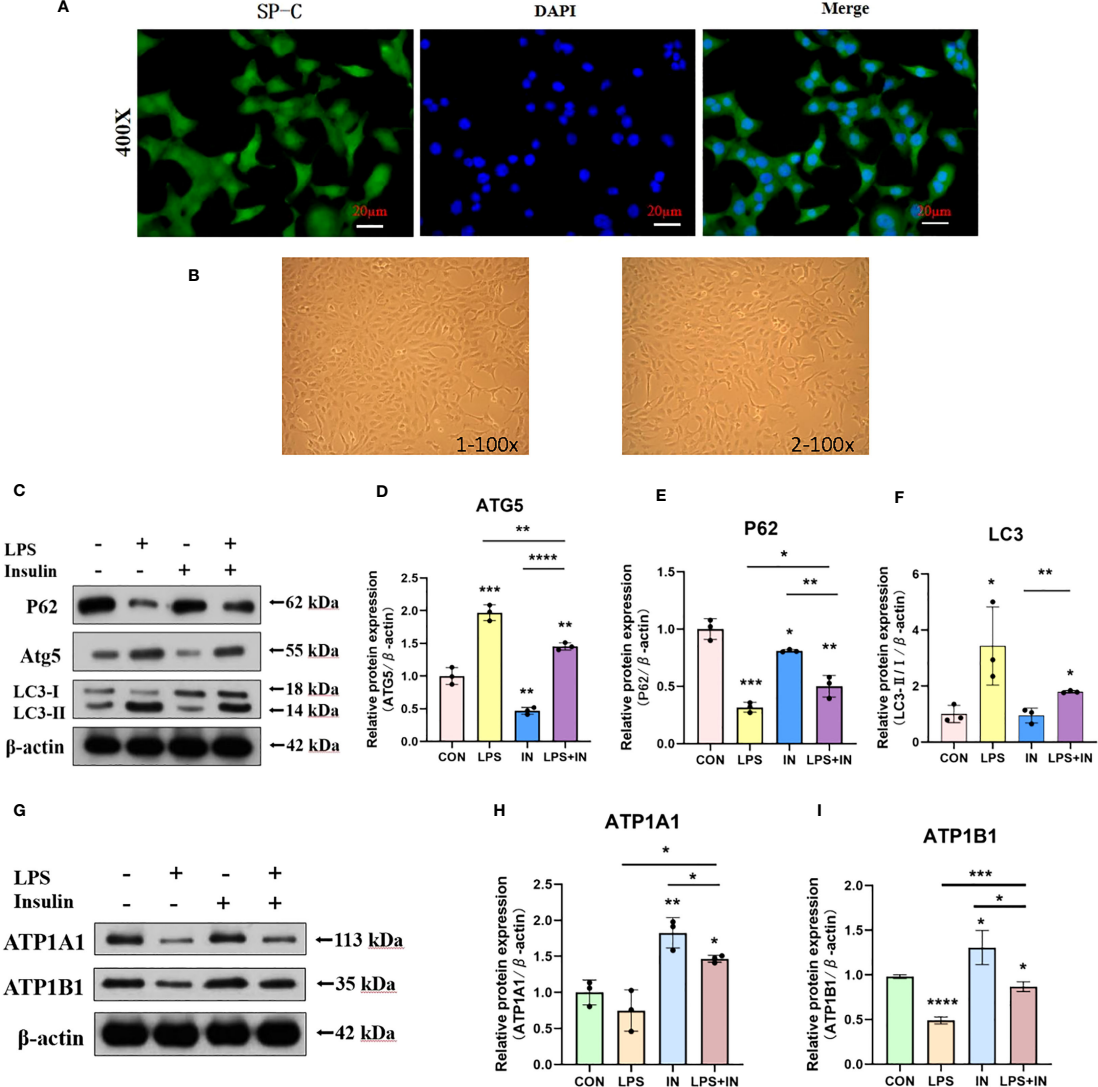
Effects of LPS and insulin on Na, K-ATPase expression and cellular activity in AT II cells. **(A)** Immunofluorescence detection of pulmonary surfactant protein SP-C, the scale bar is 20 µm, the positive signal is SP-C (green fluorescence), and the blue is the nuclear staining signal. **(B)** AT II cells were observed with an inverted phase-contrast microscope. the scale bar is 1×100 and 2×100, respectively. **(C, G)** Representative western blotting detected the levels of LC3-II/I, ATG5, P62 and ATP1A1 in AT II cells. **(D–F, H, I)** Quantitative analysis of LC3-II/I, ATG5, P62 and ATP1A1 were shown in bar graphs, respectively. The data are presented as mean ± SD. n = 3, the horizontal line represented the comparison between each two groups, *p<0.05. **p < 0.01. ***P < 0.001. ****P < 0.0001.

To ensure the accuracy, credibility, practicality and stability of our results, we also repeated and verified the above experiments on AT II cells, and to our delight is that the results are almost the same. First, we isolated AT II cells from C57 mice, purified and cultured them, and then detected pulmonary surfactant-associated protein C (SP-C) by immunofluorescence (**Figure 13A**). AT II cells grew in an island shape under an inverted phase-contrast microscope, and the cells were short spindle-shaped, polygonal or cuboidal (**Figure 13B**). Last but not least, we also induced AT II cells with LPS and intervened with insulin. The results showed that the expressions of LC3-II/I and ATG5 increased and P62 decreased after LPS administration, which suggested that LPS activated autophagy in AT II cells, while insulin played an inhibitory role on the contrary (**Figures 13C–F**). Interestingly, we detected Na, K-ATPase β1 (ATP1B1) at the same time as ATP1A1 detection, and surprisingly found that the expression trend of ATP1B1 was consistent with that of ATP1A1, both of which were inhibited by LPS and increased by insulin (**Figures 13G–I**). The above results are sufficient to prove that insulin inhibited LPS-induced autophagy activation and improved the expression of Na, K-ATPase in lung tissue, thereby limiting the generation of pulmonary edema.

## 4 Discussion

Pulmonary infection is the main cause of ARDS, especially LPS induced alveolar epithelial cell injury and increased vascular endothelial cell permeability that leading to pulmonary edema fluid leakage and thus resulting in severe hypoxemia (19). ARDS is often accompanied by abnormal Na, K-ATPase function. Upregulating Na, K-ATPase expression or increasing the amount of Na,K-ATPase may enhance trans-epithelial sodium transport in alveolar cells, thereby promoting intra-alveolar edema fluid reabsorption and limiting the development of pulmonary edema (20). The current researches focus on the mechanism of Na, K-ATPase regulatng in the alveolar epithelium during lung injury, and the dysregulation of Na, K-ATPase subsequently exacerbates the development of pulmonary edema, thus regulating alveolar epithelial Na, K-ATPase is important for limiting pulmonary edema.

Autophagy occurs in the lung is a response of alveolar epithelial cells to long-term and continuous stimuli from intrapulmonary and extrapulmonary factors (21). Liu Y et al. found that Na, K-ATPase participates in autophagy-lysomytosterios through its α1 subunit, and AMPK (one of the most important positive regulators of autophagy) and ATP1A1 may act as the “on” and “off” states of the autophagic pathways (10). In addition, the novel mechanism of ATP1A1 as a mediator of signal transduction and autophagy during ischemia-reperfusion provides new ideas for the intervention of ischemic stroke (22, 23). Our previous study revealed that the ATP1A1 regulatory mechanism may be related to the autophagy-lysosome pathway (24). Few studies have been conducted on the degradation of Na, K-ATPase via the autophagy-lysosome pathway, However, the relationship between them is worthy of extensive attention and discussions to affect the abundance and enzyme activity of ATP1A1, enhance the lung water clearance ability, and improve the prognosis of ARDS in the future.

Insulin is a normal growth hormone in human body, which has been widely used in clinical practice for a long time without any toxic side effects. It is only necessary to inject concentrated sugar to prevent the occurrence of hypoglycemia, and the dose of 6 IU/h of continuous intravenous pumped insulin is approximately the same as that of 1.0 IU/kg intraperitoneal injection in mice and 100 nM conversion in A549 and ATII cells, so the clinical results are highly consistent with the results of animal and cellular experiments. Our study was conducted to use these characteristics of insulin as described above, and validated on LPS-induced animal and cell ARDS models. We found that LPS activates autophagy and promotes inflammatory responses, leading to Na, K-ATPase degradation and alveolar epithelial damage thus exacerbating intra-alveolar fluid accumulation. In contrast, insulin inhibited both autophagy and inflammatory response, allowing Na, K-ATPase activity to be improved, thus reversing the impaired lung water clearance caused by LPS in ARDS. It is highly likely that there are many close links between the key factors of autophagy, inflammation and Na, K-ATPase, and insulin plays a crucial role in this regard. The specific mechanisms deserve to be explored in depth.

Several key autophagy proteins were well validated in our experiments. P62/SQSTM1, is a multifunctional autophagy protein. It is involved in ubiquitin-proteasome and autophagy-lysosome degradation processes and is an important regulatory molecule linking ubiquitinated proteins to the autophagic machinery (13). It is now known that insulin activates mTORC1 through upregulation of AKT to elevate P62, which binds to Keap1 and transports it to the lysosome for degradation, where Keap1-bound transcription factor NRF2 dissociates from Keap1 into the nucleus and initiates transcription for anti-inflammatory effects (25). We found that insulin also inhibited autophagy and upregulated P62 levels in ARDS mice and A549 cells as well as ATII cells. The above evidence suggests that insulin is likely to upregulate P62 levels, inhibit the release of inflammatory factors, and reduce inflammatory cell infiltration and thus reduce pulmonary water production. Interestingly, in our previous study, we found that ATP1A1 interacts with P62 through Co-IP and western blot verification (24). It is thought-provoking that P62 may act as a key regulatory mediator in this, so we conjecture that insulin most likely regulates ATP1A1 and P62 expression through inhibition of autophagy and thereby plays some key role in the accumulation of alveolar epithelial fluid.

Inflammatory cytokines are involved in the control of autophagy and known to play a key role in the development of ARDS. In turn, autophagy regulates inflammatory factors. TNF-α is the earliest endogenous mediator and amplifies the inflammatory response and the production of IL-1β leads to lung epithelial injury (26). Insulin inhibits the release of pro-inflammatory factors such as TNFα and IL-1β, and increases the production of anti-inflammatory factors such as IL-10 (27). Autophagy reduces IL-1β secretion by inhibiting inflammasome activation (28). Activation of autophagy inhibits TNF-α secretion (29). Yang S et al. found that decreased hepatocyte autophagy promotes IL-1β/TNF-induced necrosis from impaired energy homeostasis and lysosomal permeabilization and inflammation through the secretion of exosomal damage-associated molecular patterns (30). It is dependent on the PI3K/AKT/mTOR signalling pathway to promote autophagy and suppresses inflammation in TNF-α-treated keratinocytes and psoriatic mice (31). Based on these reports and our previous results, the specific mechanisms of insulin in inhibitng autophagy and inflammation deserve further exploration.

Nevertheless, there are some conflicting limitations to our study. In formal clinical care, some clinical observations and experimental animal studies suggest that it is uncertain whether insulin prevents the development of ARDS, and studies have shown that in diabetes with systemic inflammatory, insulin exacerbates the production of pro-inflammatory mediators and inhibits cardiac function (32). There are much debates as to whether insulin is harmful or beneficial in ARDS. What is certain, however, is that the benefits of the right dose of insulin are more pronounced in patients with ARDS. In addition, it is known that autophagy has a dual roles in different models. The exact mechanism needs to be further investigated.

Zhang et al. reported that chloroquine, a chemical inhibitor of autophagy, and silencing of Atg7 attenuated LPS-induced autophagosome formation in human pulmonary microvascular endothelial cells, reduced cell viability, and aggravated LPS-induced monolayer permeability (11). This is contrary to our conclusion. It has also been reported that neutrophil autophagy is increased in LPS-induced mice, which leading to increased release of granule contents. Knockdown of the autophagy gene Atg5 abolishes the promotion of MPO by LPS and the chemokine fMLP, which limits the release of granule contents and thereby alleviates ARDS (33). Thus, autophagy appears to contribute to endothelial barrier disruption caused by edema-inducing agents and also plays an important role in acute lung injury, and inhibition of autophagy can protect against endothelial barrier function caused by ARDS. To address these phenomena, it is necessary to further investigate the effects of endothelial cell-specific genetic alterations in ARDS.

## 5 Conclusion

In summary, Our experiments were conducted in LPS-induced ARDS, with a view to investigating the impairment of AFC in ARDS. We first used the vivo experiments to confirm the effect of insulin inhibition of autophagy on alveolar epithelial damage and AFC under LPS-induced conditions. This was followed by functional reversion experiments, which were further confirmed in LPS-induced ARDS A459 cells and ATII cells. The aim is to elucidate the possible mechanisms by which insulin regulates Na, K-ATPase α1 and inflammatory response through inhibiting autophagy, thereby reverses the impaired AFC in LPS-induced ARDS. However, our studies are only preliminary, revealing the effects of insulin on Na, K-ATPase α1 and autophagy proteins expression and inflammatory responses. This work will provide a partial theoretical and experimental basis for the course and clinical treatment of ARDS by elucidating the biological functions of insulin and relevant autophagic markers in the field of lung protection in ARDS.

## Data availability statement

The datasets presented in this study can be found in online repositories. The names of the repository/repositories and accession number(s) can be found below: PXD040288 (ProteomeXchange).

## Ethics statement

All experimental animal treatment procedures were approved by the Department of Laboratory Animals of Xiangya School of Medicine, Central South University, and are carried out under the Guide for the Use of Laboratory Animals by the National Institutes of Health.

## Author contributions

Q-QW designed the study, X-PW and Q-QW conducted the data analysis, and wrote the manuscript. X-PW and Q-QW participated in and contributed to the experiments of this study. X-PW, ML, R-QZ participated in manuscript revision. All authors read and approved the final manuscript.
