# Supplementary figures and images for: Insulin reverses impaired alveolar fluid clearance in ARDS by inhibiting LPS-induced autophagy and inflammatory
Source: Front Immunol. 2023 Aug 15;14:1162159. doi: 10.3389/fimmu.2023.1162159 (PMC10466042; doi:10.3389/fimmu.2023.1162159)

Z-score

2  
1  
0  
-1  
-2

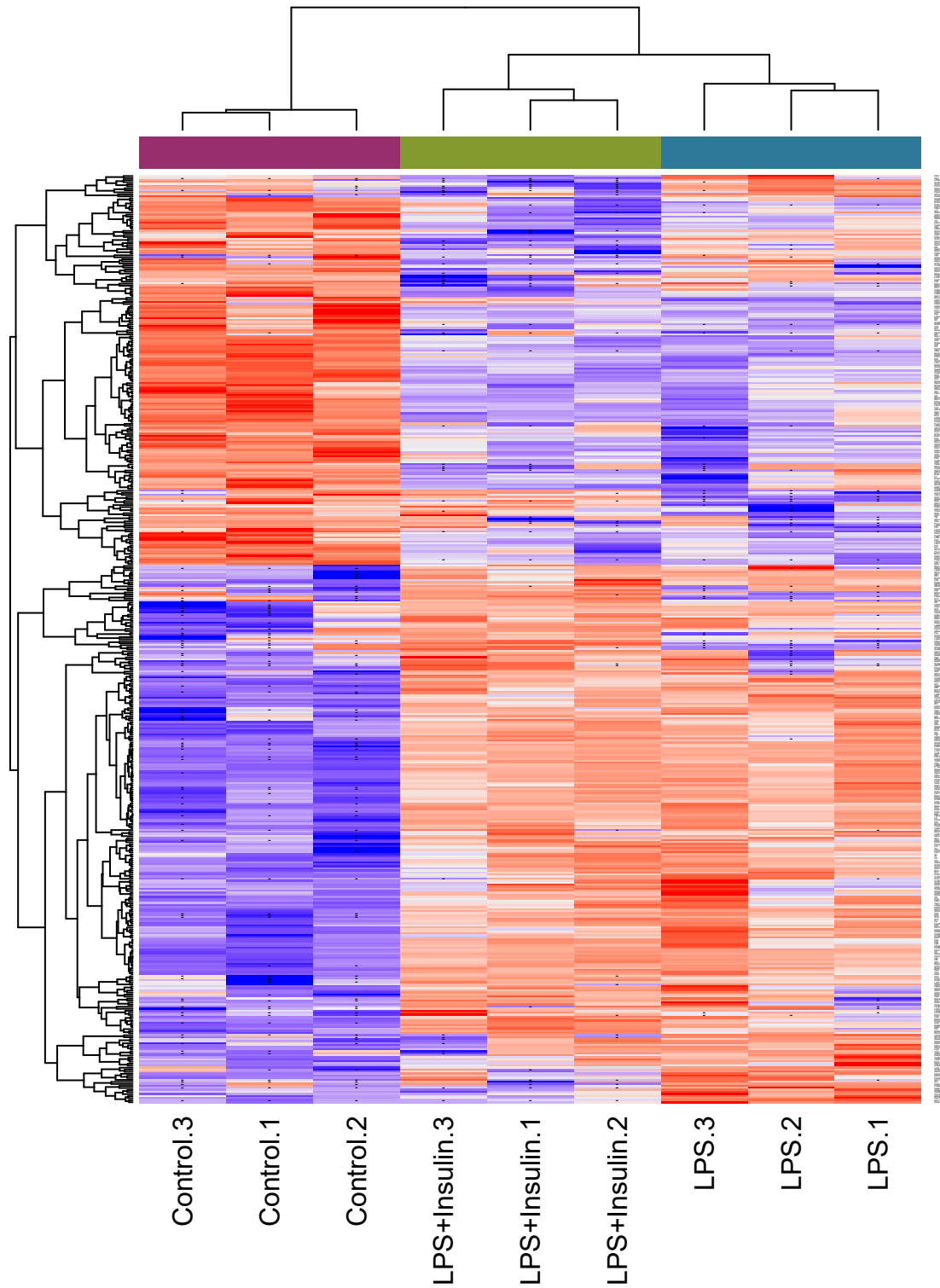

Bio.Group

- LPS+Insulin
- LPS
- Control

Supplement: Supplementary file 1 [file DataSheet_1.zip › all-cluster-1.pdf]

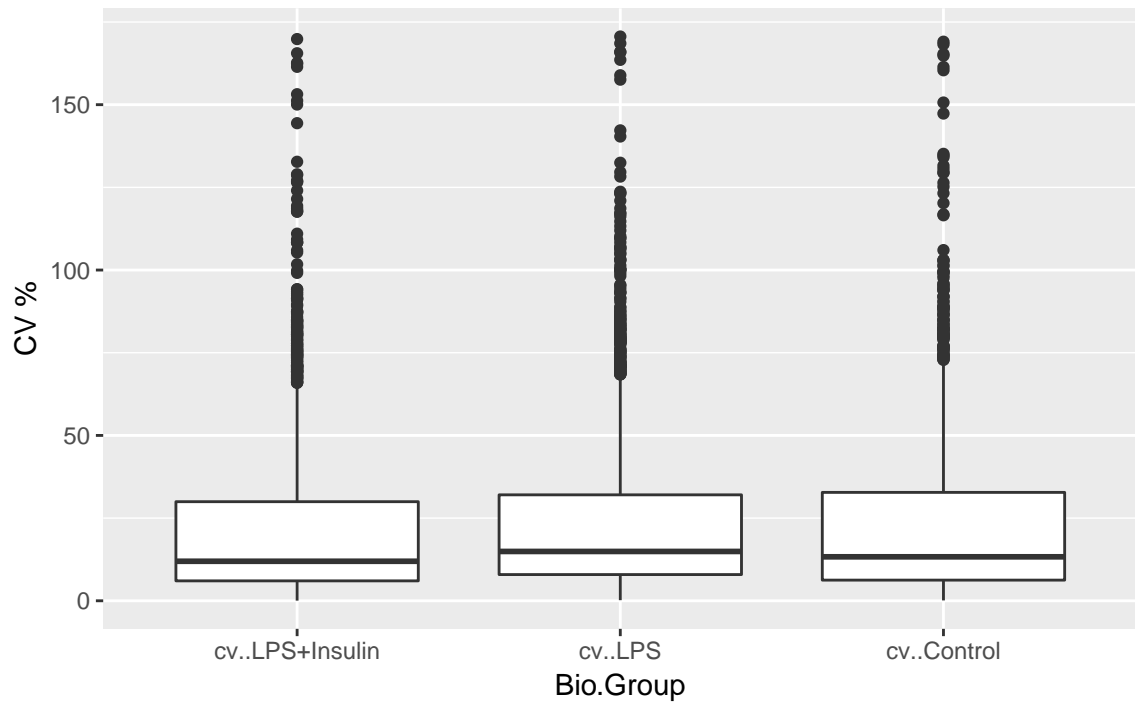

Supplement: Supplementary file 1 [file DataSheet_1.zip › CV.pdf]

Z-score

2  
1  
0  
-1  
-2

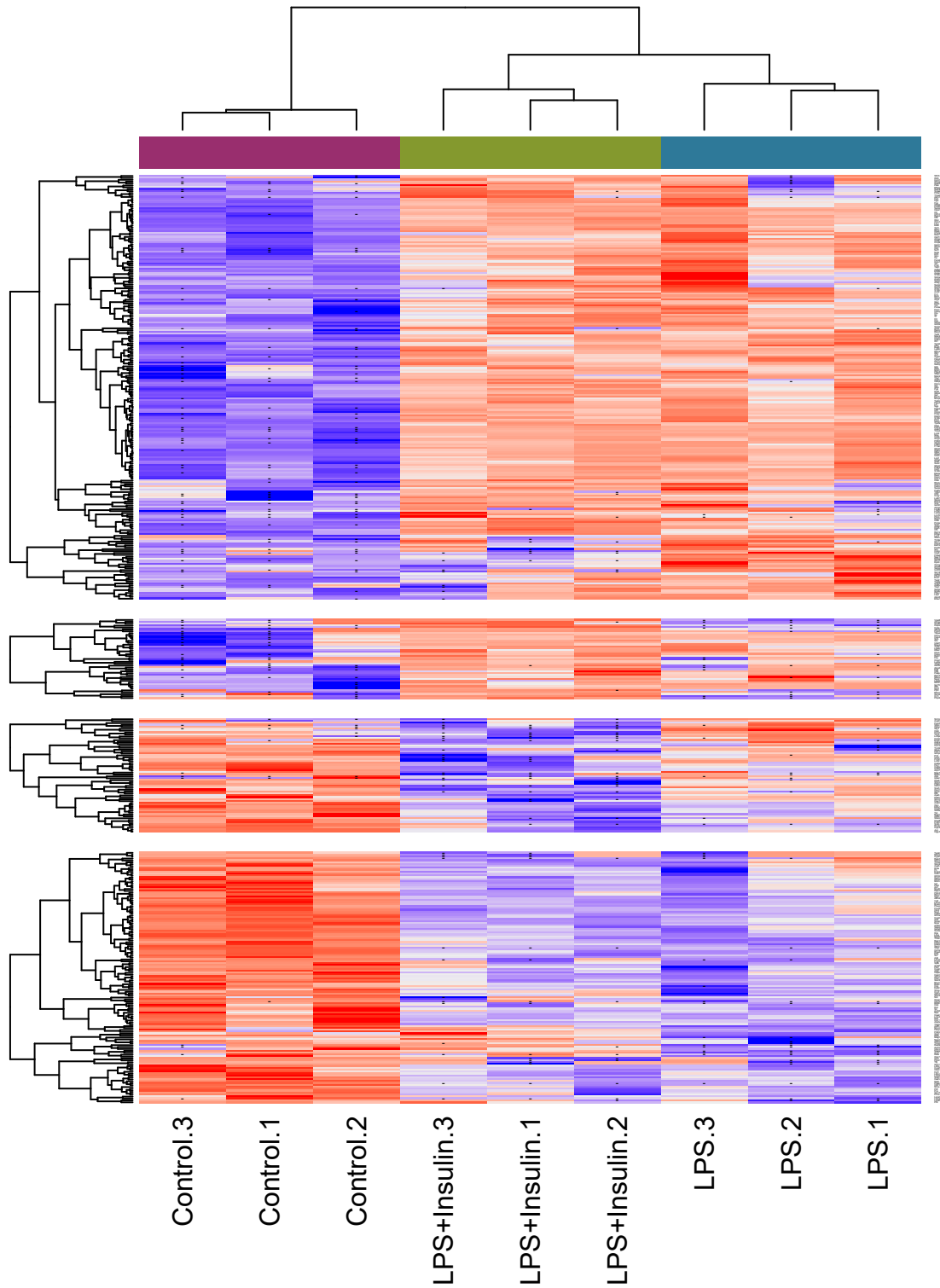

Bio.Group

- LPS+Insulin
- LPS
- Control

Supplement: Supplementary file 1 [file DataSheet_1.zip › figure-latex/all-cluster-2.pdf]

# Protein Abundance Range

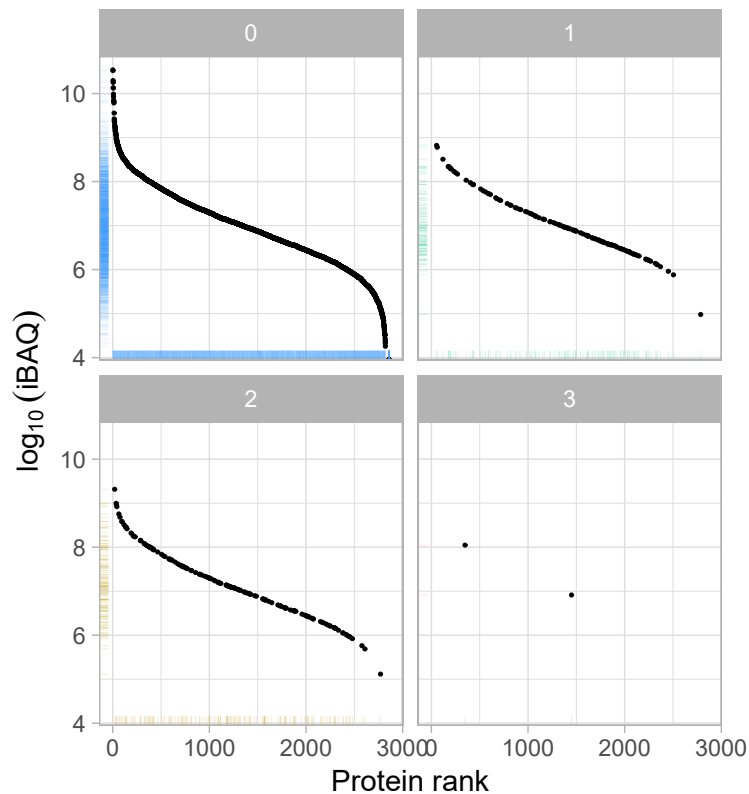

Supplement: Supplementary file 1 [file DataSheet_1.zip › figure-latex/all-ranking-1.pdf]

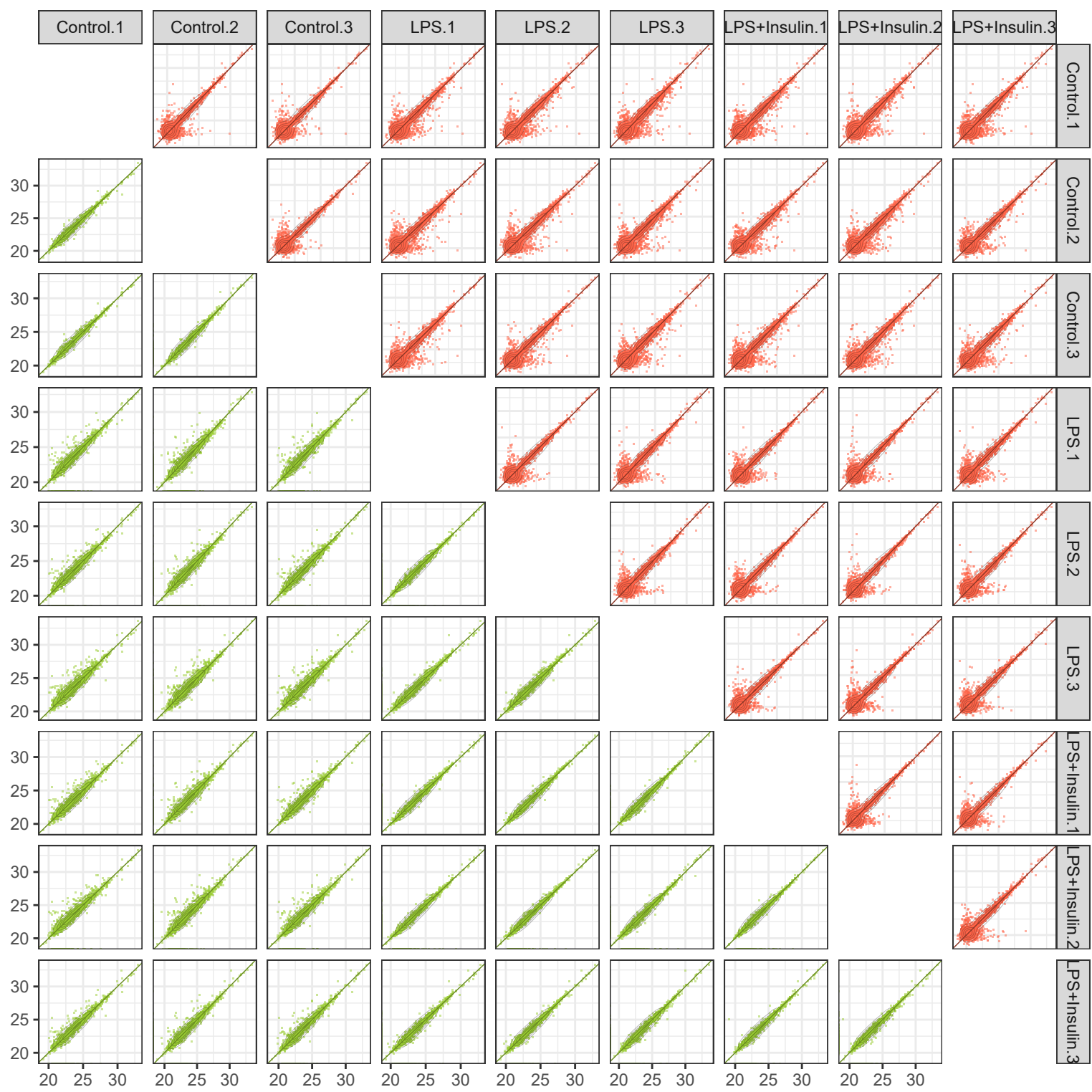

Supplement: Supplementary file 1 [file DataSheet_1.zip › figure-latex/correlation-1.pdf]

# LPS--Control

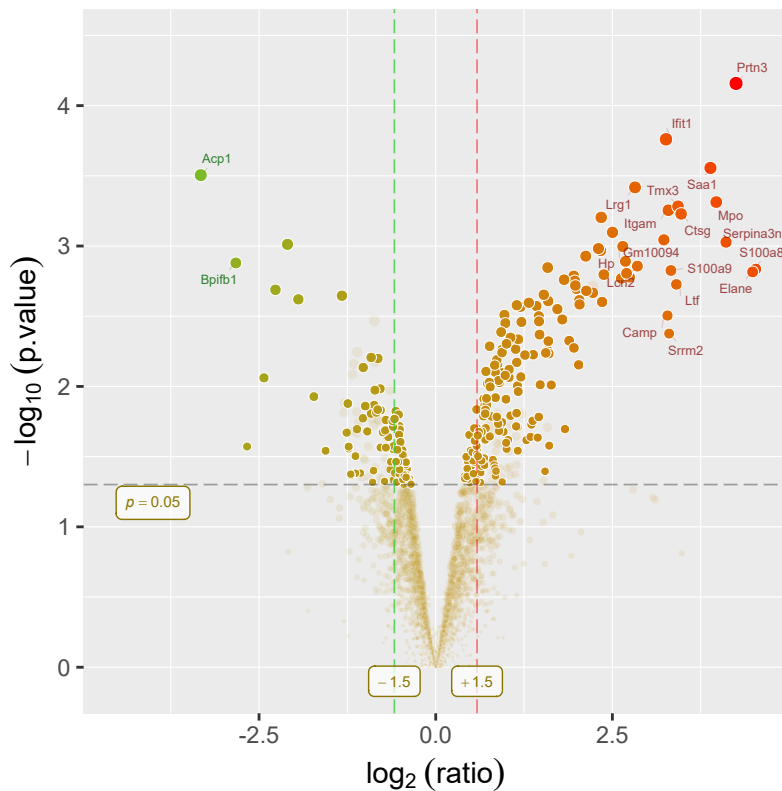

## Significant legend

- Down-regulated
- Not-significant
- Up-regulated

Supplement: Supplementary file 1 [file DataSheet_1.zip › LPS--Control-volcano-fc-1.pdf]

# LPS+Insulin--Control

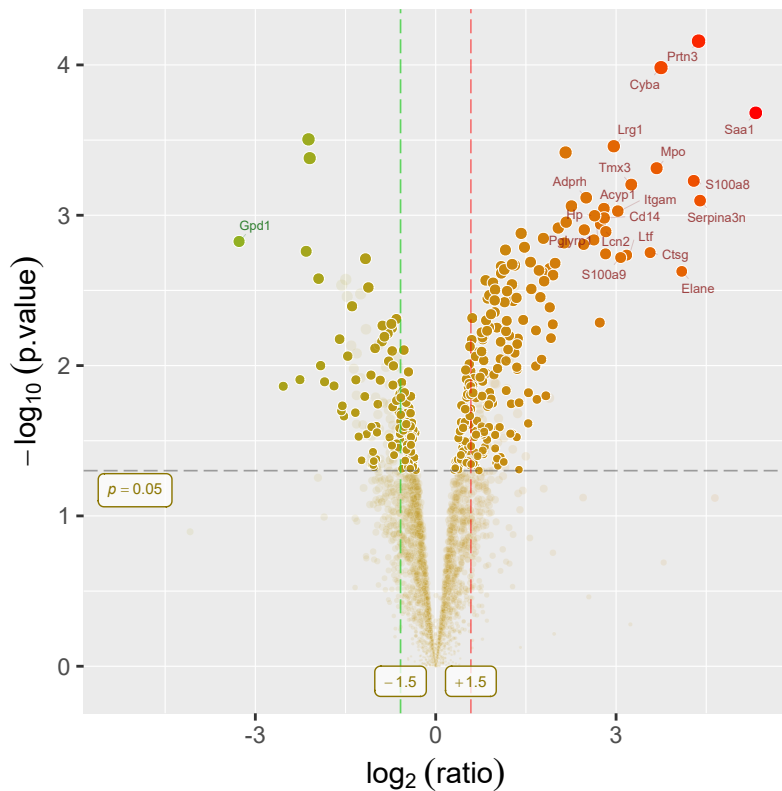

## Significant legend

- Down-regulated
- Not-significant
- Up-regulated

Supplement: Supplementary file 1 [file DataSheet_1.zip › LPS-Insulin--Control-volcano-fc-1.pdf]

# LPS+Insulin--LPS

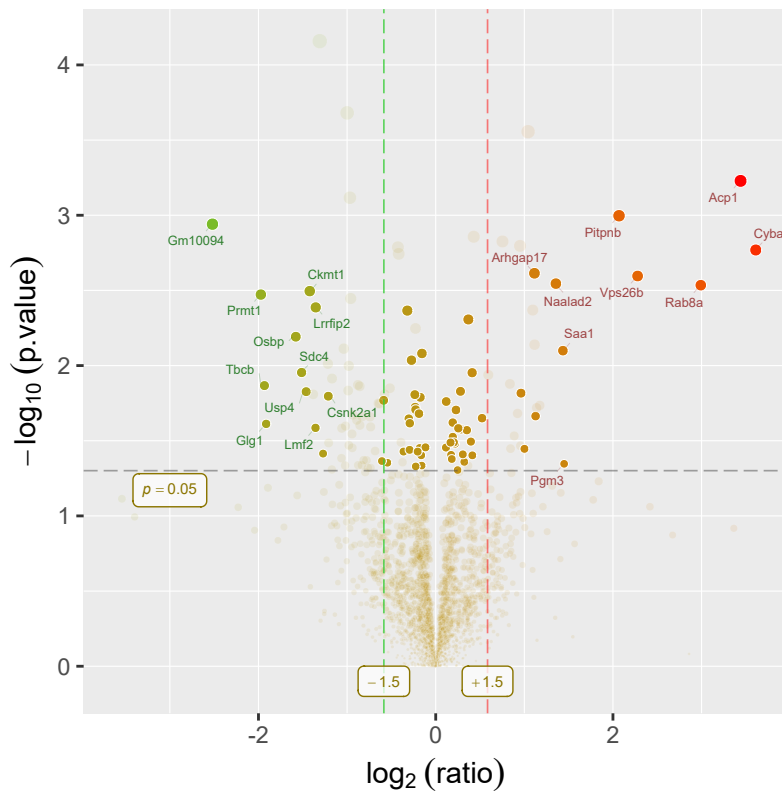

## Significant legend

- Down-regulated
- Not-significant
- Up-regulated

Supplement: Supplementary file 1 [file DataSheet_1.zip › LPS-Insulin--LPS-volcano-fc-1.pdf]

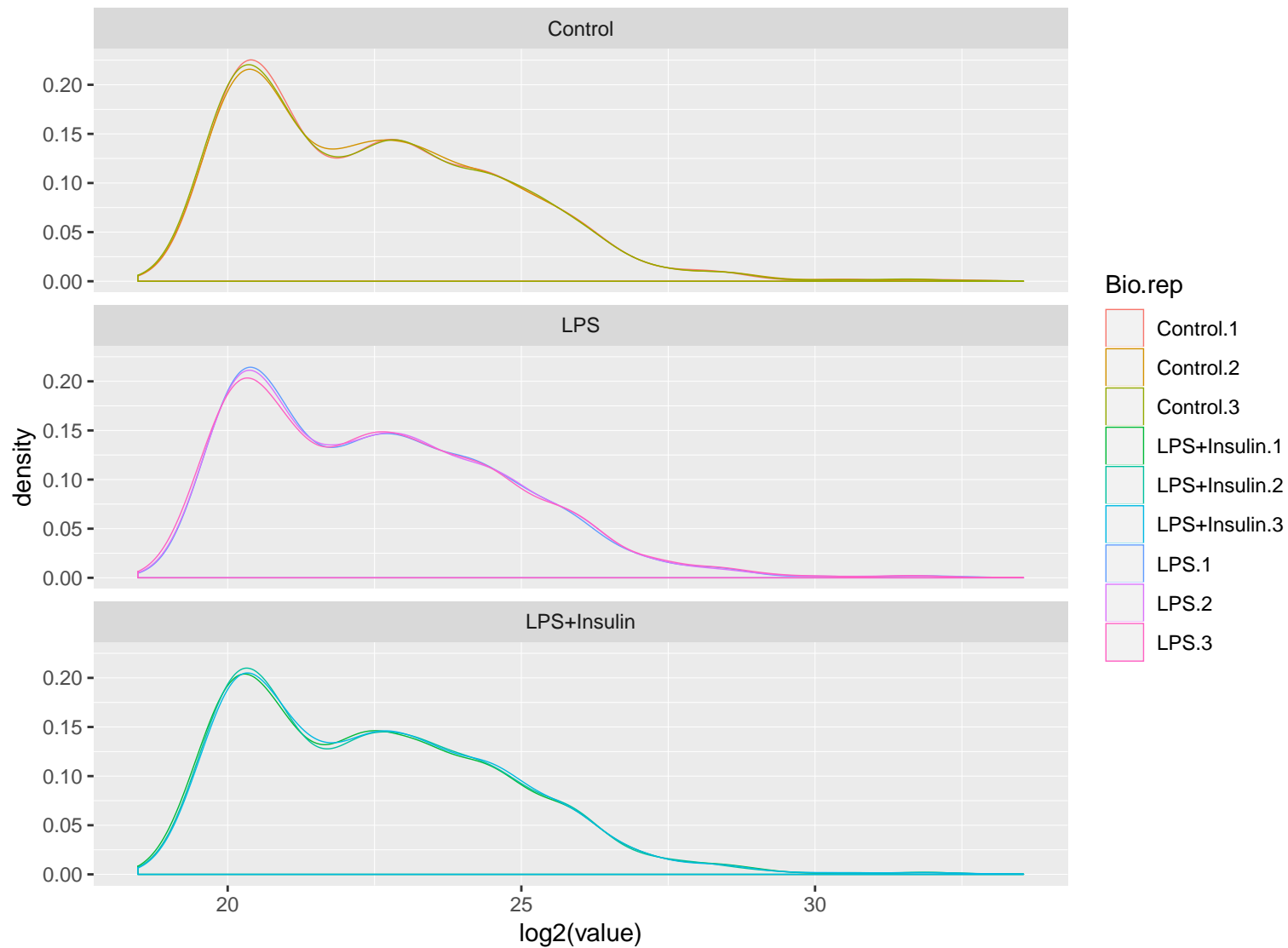

Supplement: Supplementary file 1 [file DataSheet_1.zip › norm_smry.pdf]

| Sample ID | Conc.(ug/ml) | Volume(ml) | Total（ug） |
| --- | --- | --- | --- |
| C1 | 1435.11 | 1 | 1435.11 |
| C2 | 1290.579 | 1 | 1290.579 |
| C3 | 2238.06 | 1 | 2238.06 |
| L1 | 1595.7 | 1 | 1595.7 |
| L2 | 2800.125 | 1 | 2800.125 |
| L3 | 2671.653 | 1 | 2671.653 |
| I1 | 1932.939 | 1 | 1932.939 |
| I2 | 2350.473 | 1 | 2350.473 |
| I3 | 2125.647 | 1 | 2125.647 |

**
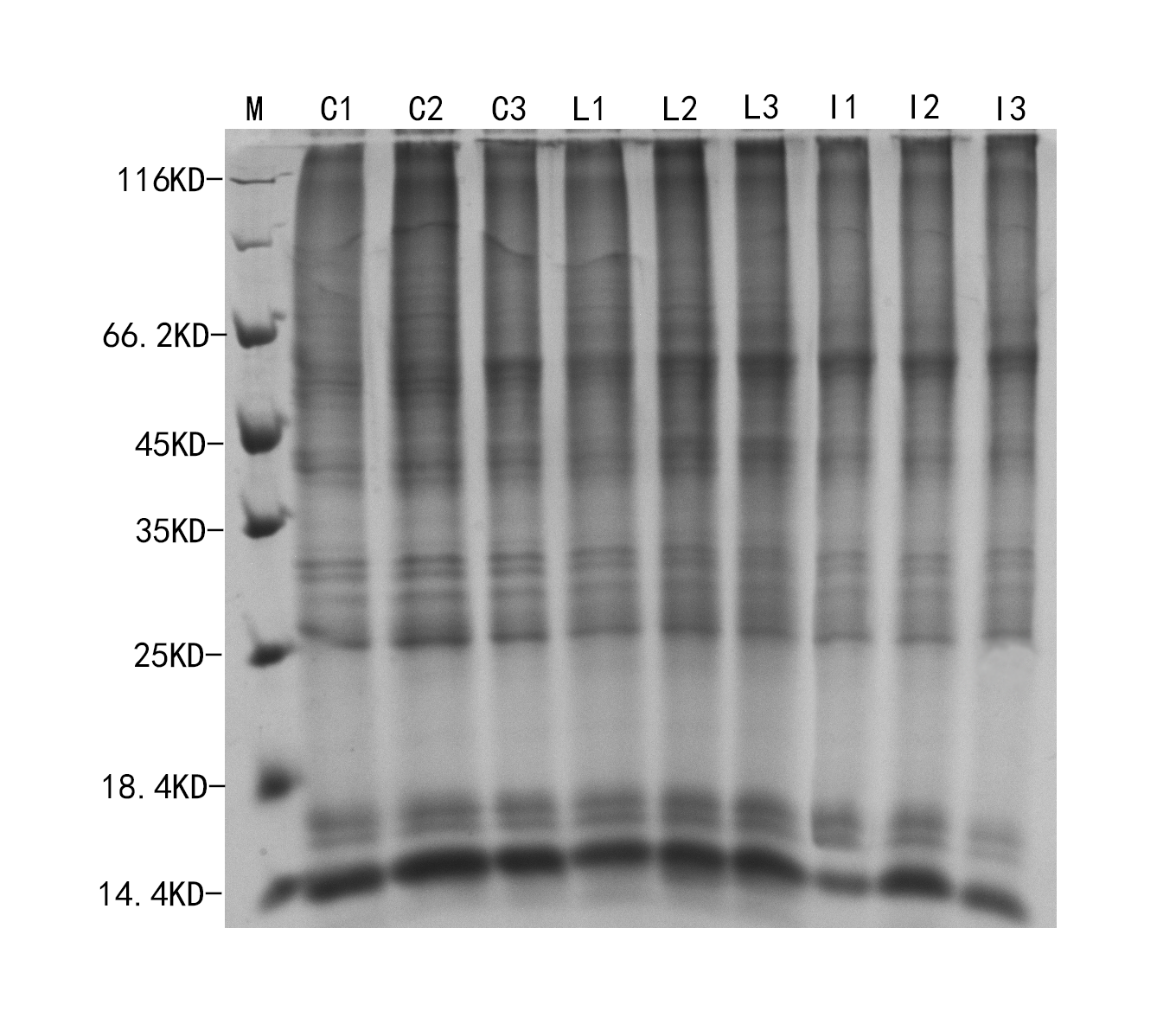
**

**
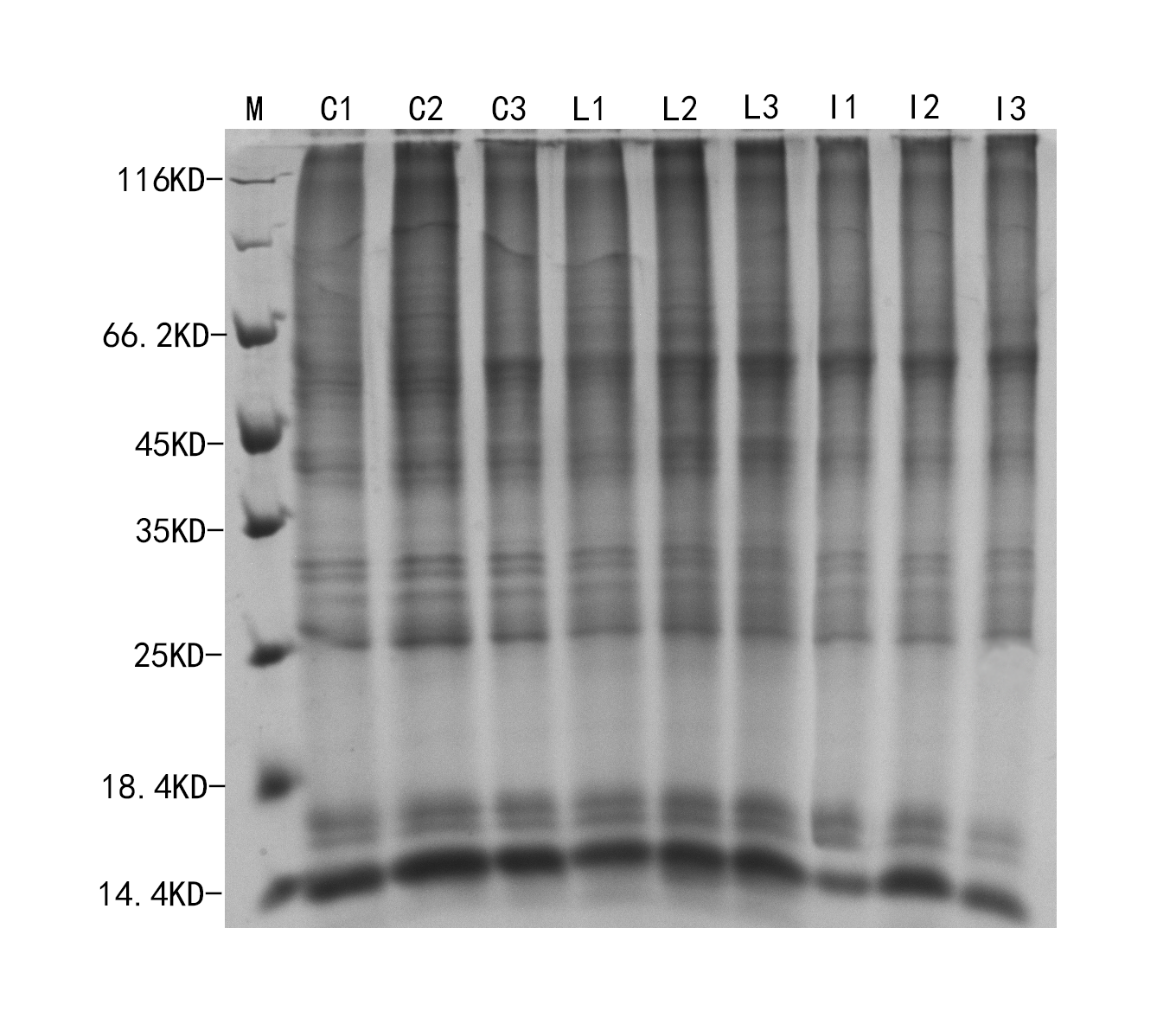
**

Supplement: Supplementary file 1 [file DataSheet_1.zip › QC.docx]

## Slide 1
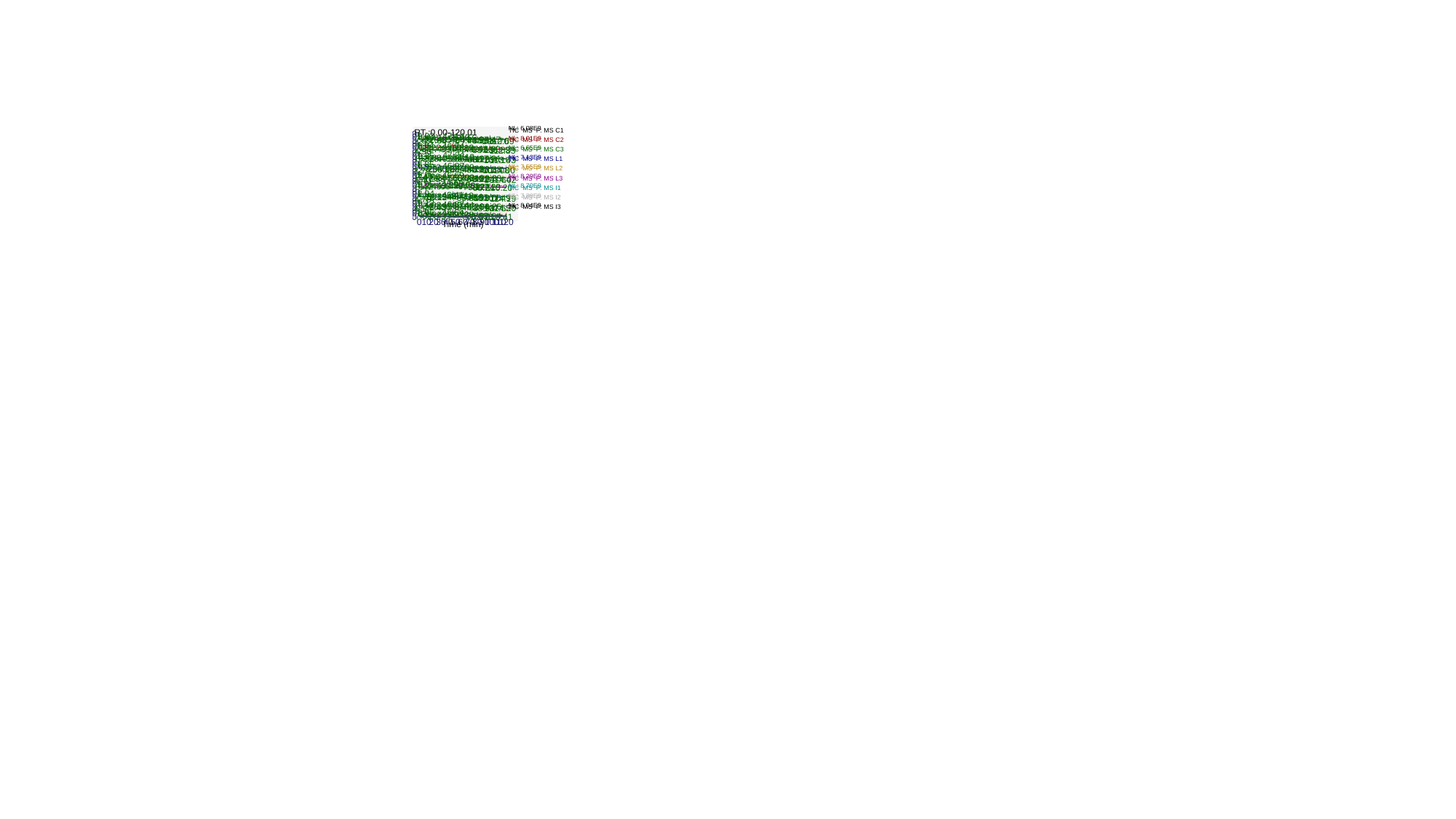

Supplement: Supplementary file 1 [file DataSheet_1.zip › TIC.pptx]
