# Supplementary material for: Insulin reverses impaired alveolar fluid clearance in ARDS by inhibiting LPS-induced autophagy and inflammatory
Source: Front Immunol. 2023 Aug 15;14:1162159. doi: 10.3389/fimmu.2023.1162159 (PMC10466042; doi:10.3389/fimmu.2023.1162159)

**Figure 9**. **Effects of LPS and insulin on Na, K-ATPase expression and cellular activity in A549 cells**. (A)(B)(E)(F) Representative western blotting detected the levels of ATP1A1 in A549 cells by different LPS and insulin concentrations and duration (C)(D)(H)(I) Quantitative analysis of ATP1A1 was shown in bar graphs, respectively. (I)(J) Effects of different doses of LPS and insulin on the survival rate of A549 cells using CCK-8 assay. The data are presented as mean ± SD. n = 3, the horizontal line represented the comparison between each two groups , **p*＜0.05. ***p* < 0.01. *** *p* < 0.001. **** *p* < 0.0001

**LPS concentrations**


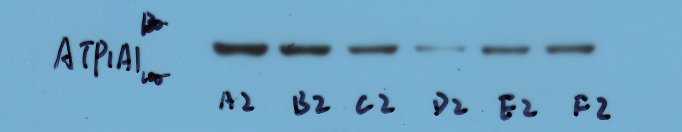


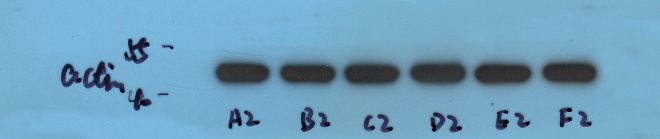


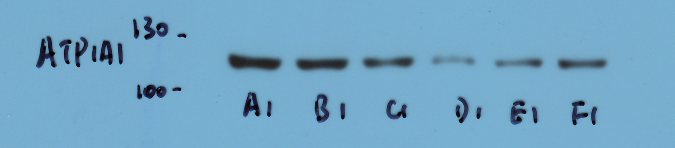


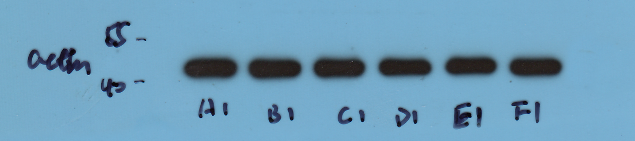


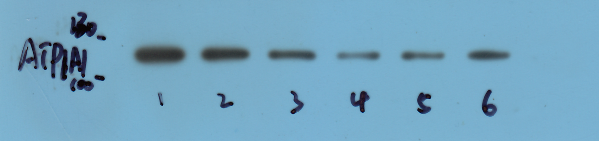


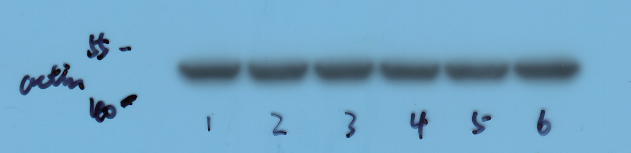


**LPS duration**


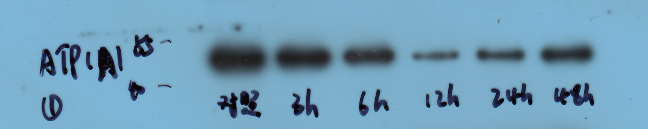


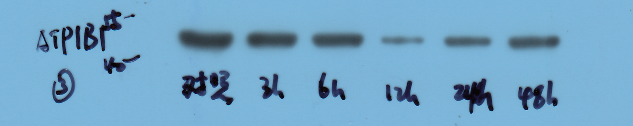


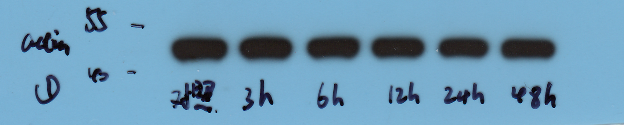


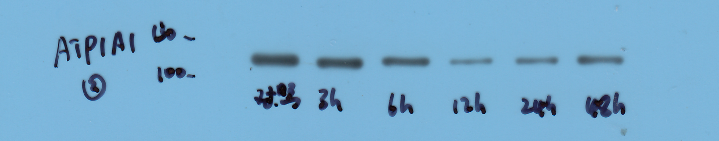

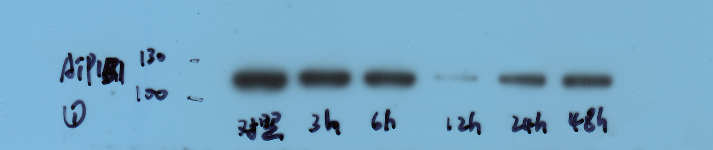


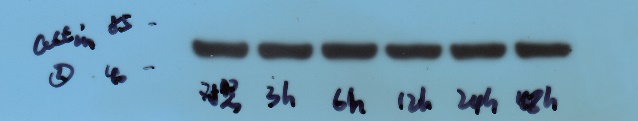


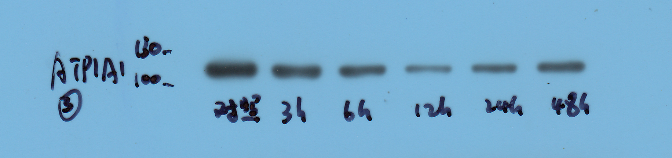


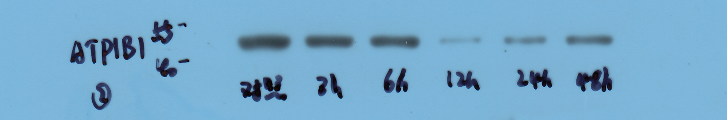


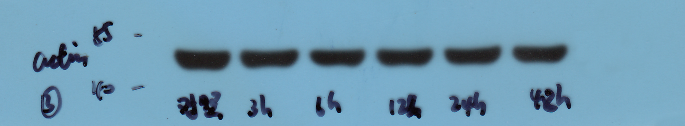


**insulin concentrations**


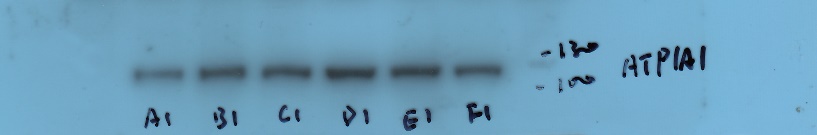


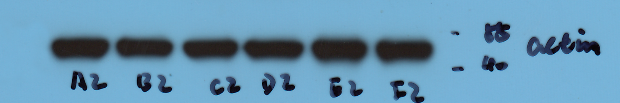


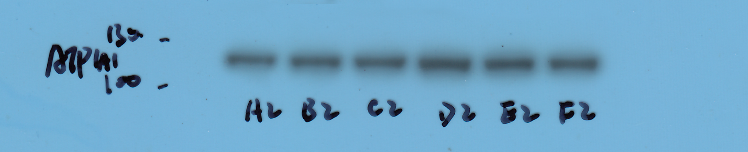


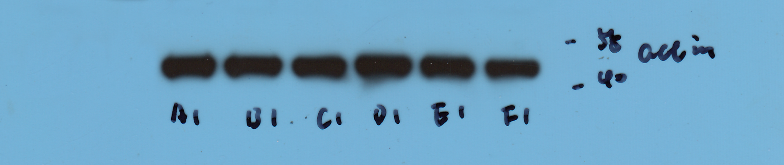


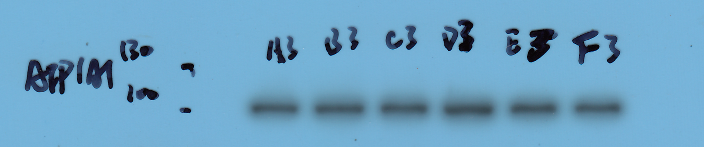


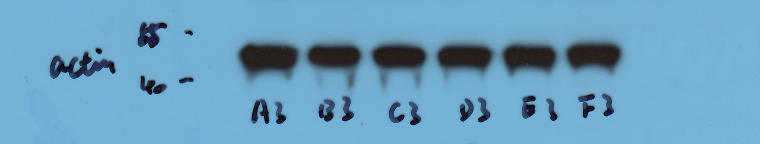


**insulin duration**


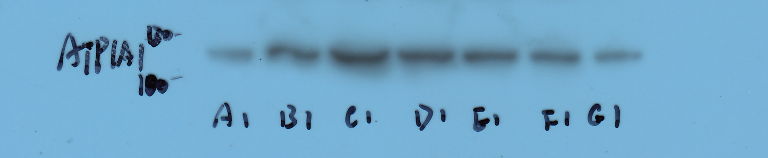


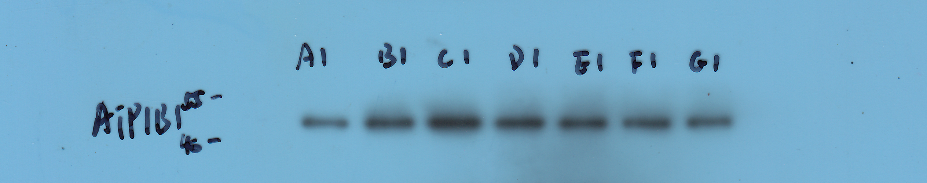

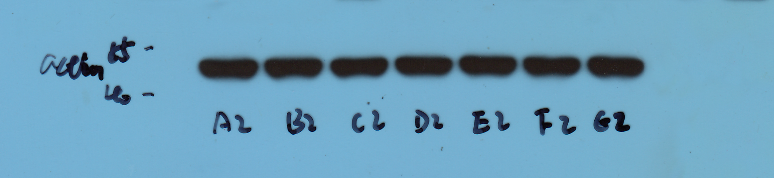


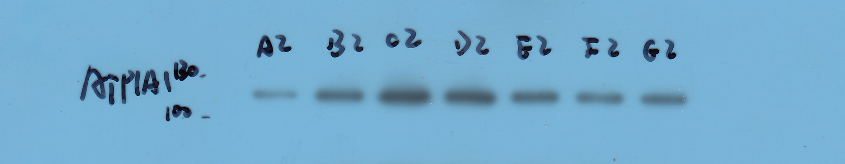


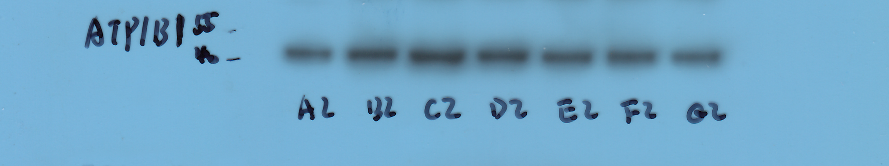

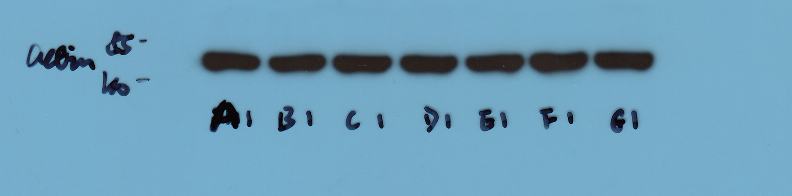


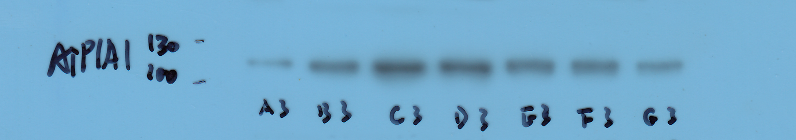


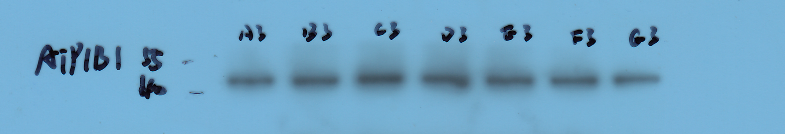

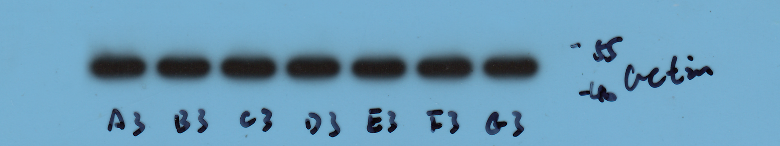


**Figure 10**. **Effects of insulin on autophagy-related proteins and ATP1A1 expression in LPS-induced A549 cells**. (A) Representative western blotting detected the levels of LC3-II/I, ATG5, Beclin-1, P62 and ATP1A1 (B)(C)(D)(E)(F) Quantitative analysis of LC3-II/I, Beclin-1, ATG5, P62 and ATP1A1 were shown in bar graphs, respectively. The data are presented as mean ± SD. n = 3, the horizontal line represented the comparison between each two groups, **p*＜0.05. ***p* < 0.01. ****P* < 0.001. *****P*< 0.0001.

.

**ATP1A1**


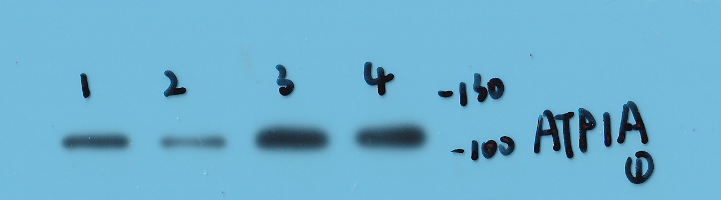


**P62**


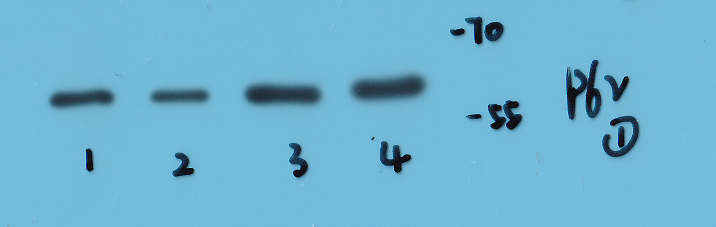


**ATG5**


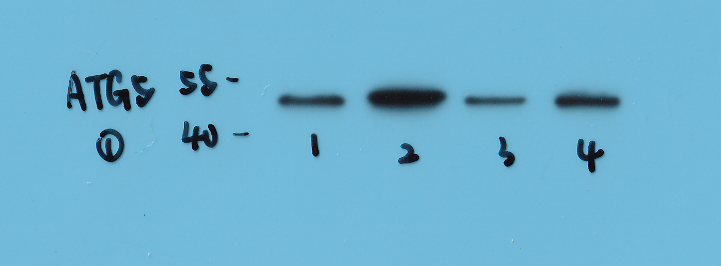


**BECLIN-1**


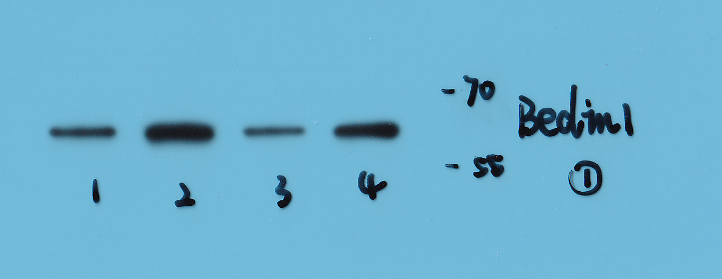


**LC3**


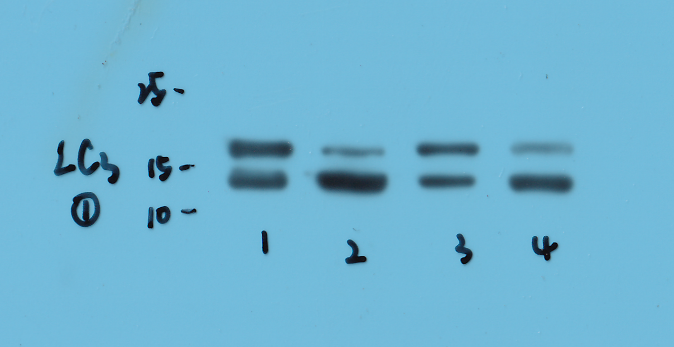


**ACTIN**


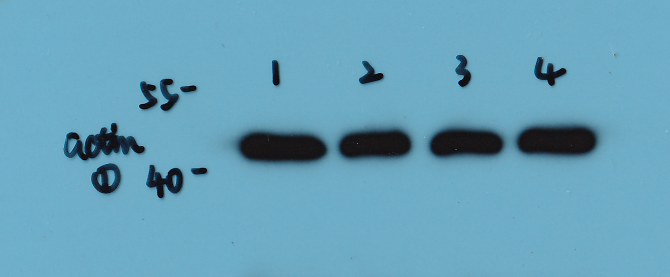


**ATP1A1**


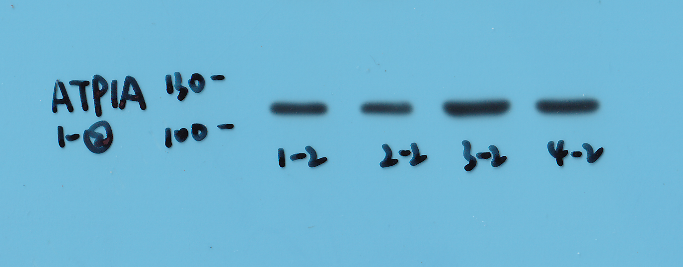


**P62**


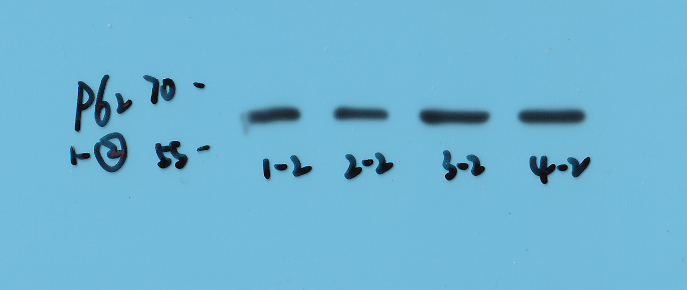


**ATG5**


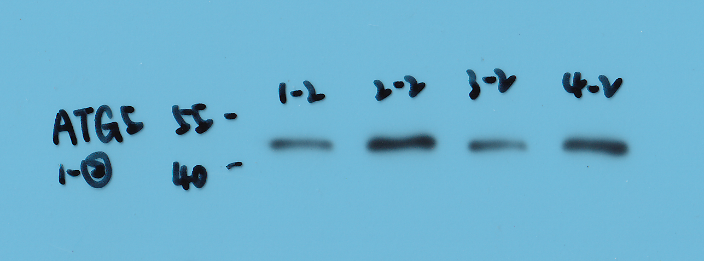


**BECLIN-1**


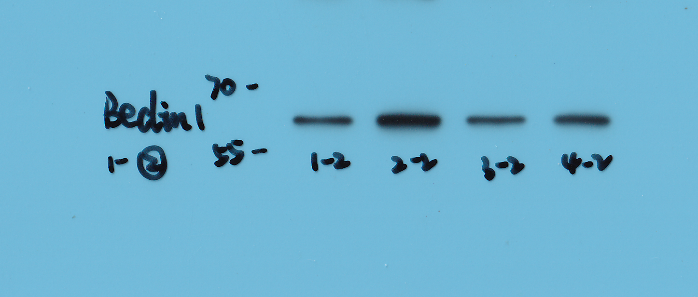


**LC3**


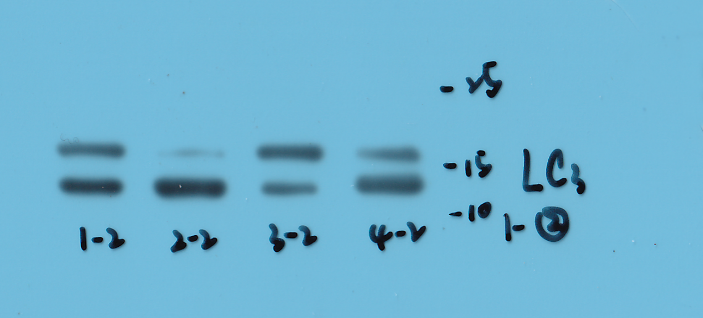


**ACTIN**


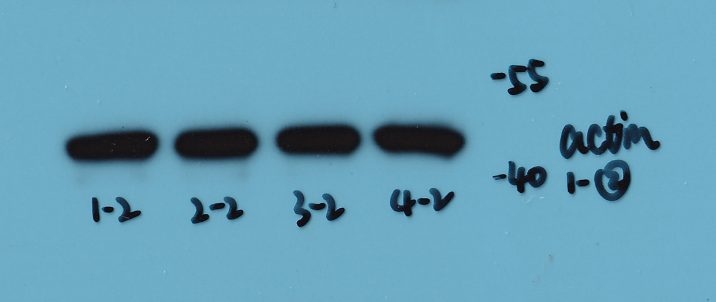


**ATP1A1**


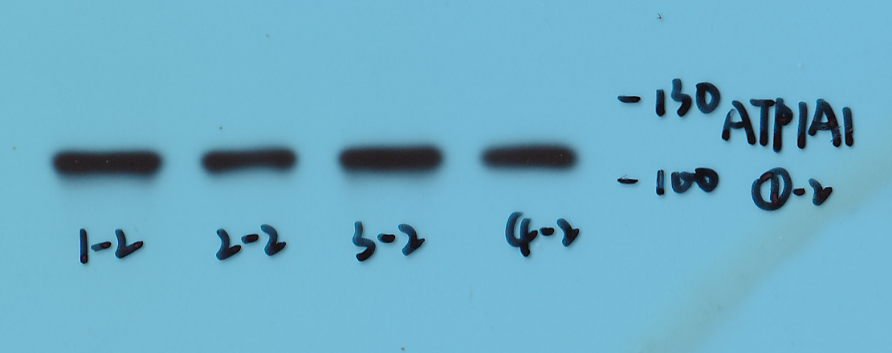


**P62**


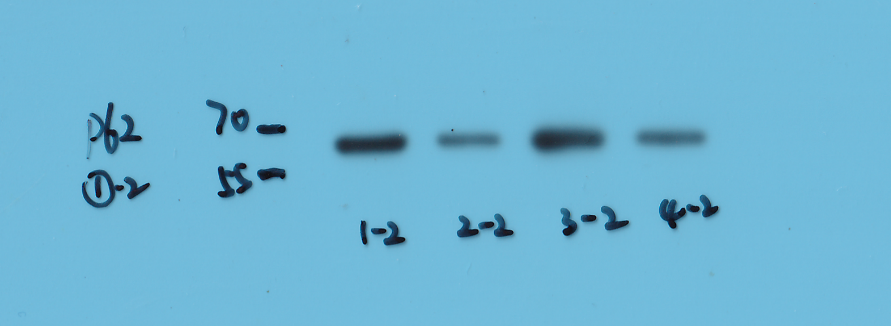


**ATG5**


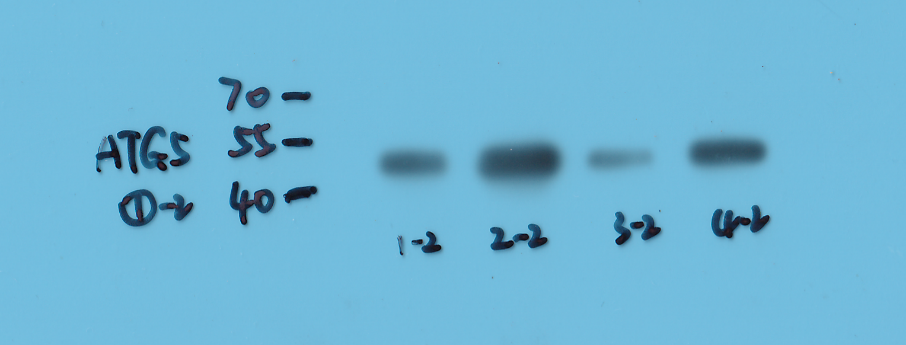


**LC3**


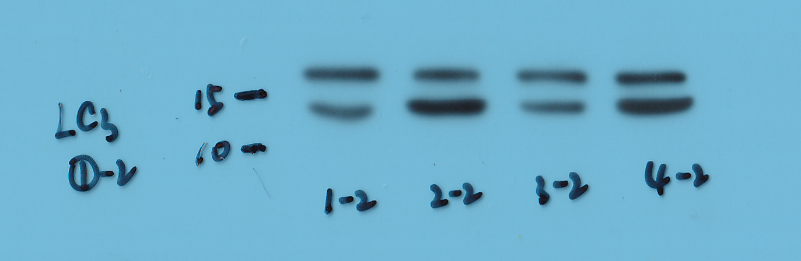


**ACTIN**


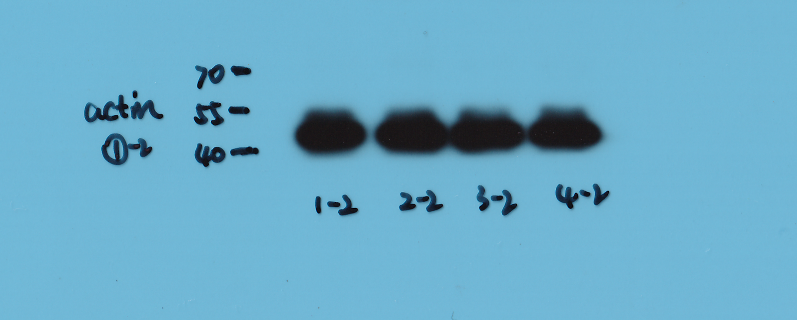


**Figure 13**. **Effects of LPS and insulin on Na, K-ATPase expression and cellular activity in AT II cells**. (A) Immunofluorescence detection of pulmonary surfactant protein SP-C, the scale bar is 20 µm, the positive signal is SP-C (green fluorescence), and the blue is the nuclear staining signal. (B) AT II cells were observed with an inverted phase-contrast microscope. the scale bar is 1×100 and 2×100, respectively. (C)(G) Representative western blotting detected the levels of LC3-II/I, ATG5, P62 and ATP1A1 in AT II cells. (D)(E)(F)(H)(I) Quantitative analysis of LC3-II/I, ATG5, P62 and ATP1A1 were shown in bar graphs, respectively. The data are presented as mean ± SD. n = 3, the horizontal line represented the comparison between each two groups, **p*＜0.05. ***p* < 0.01. ****P* < 0.001. *****P*< 0.0001.

.

**P62**


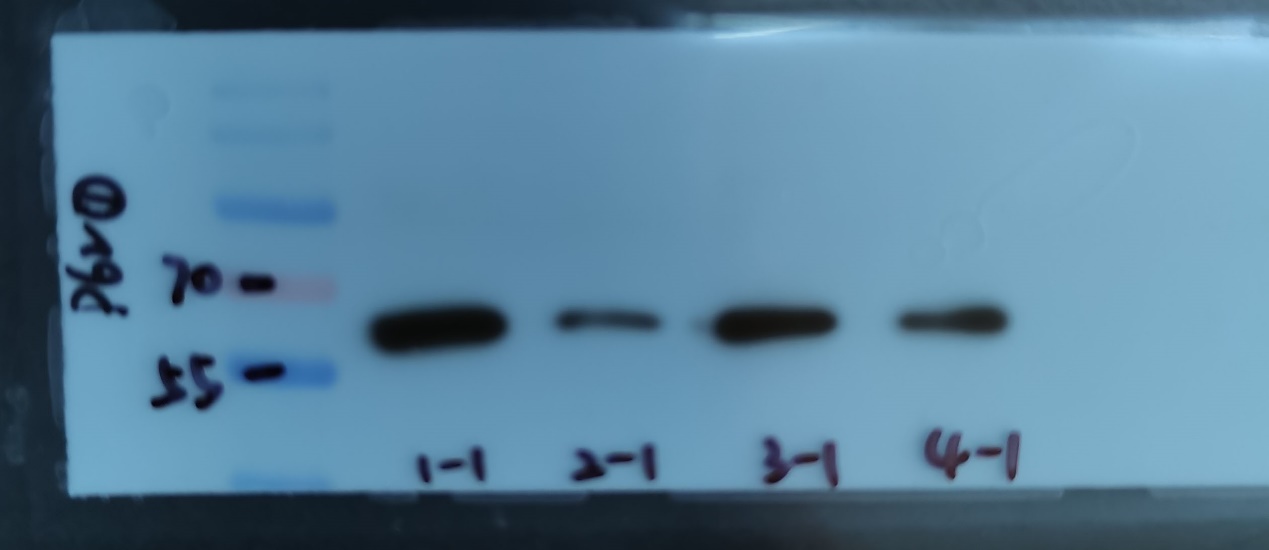


**ATG5**


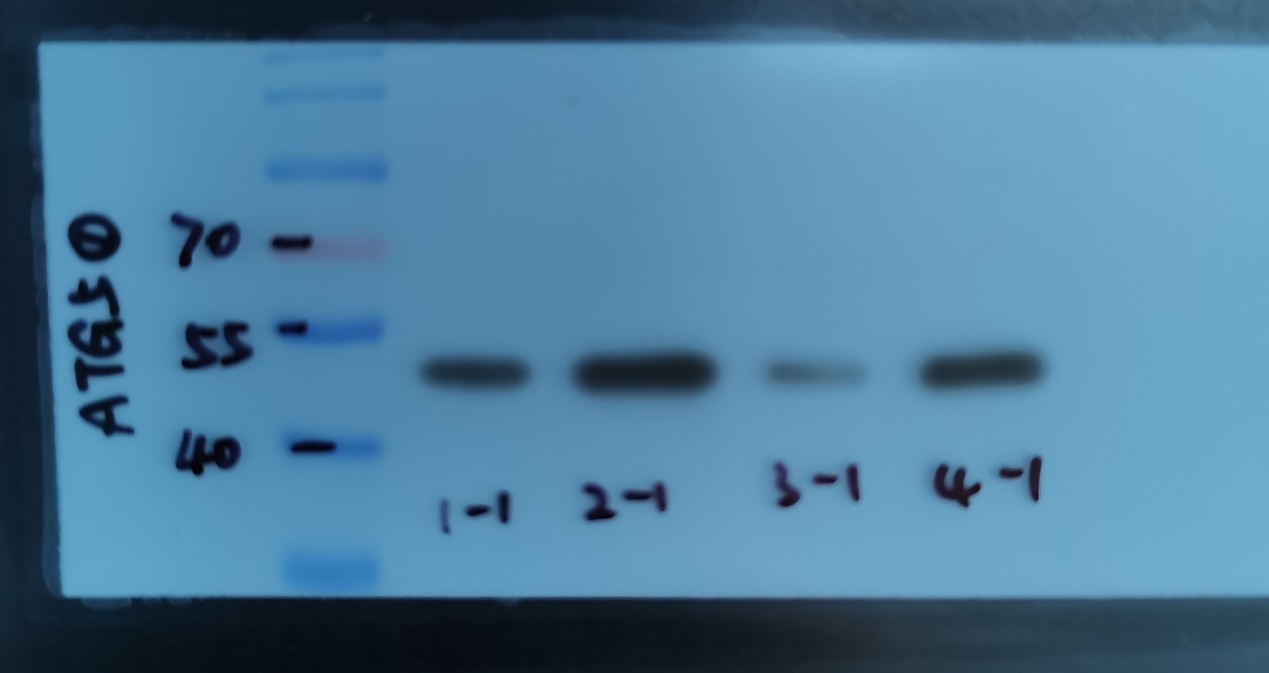


**LC3**


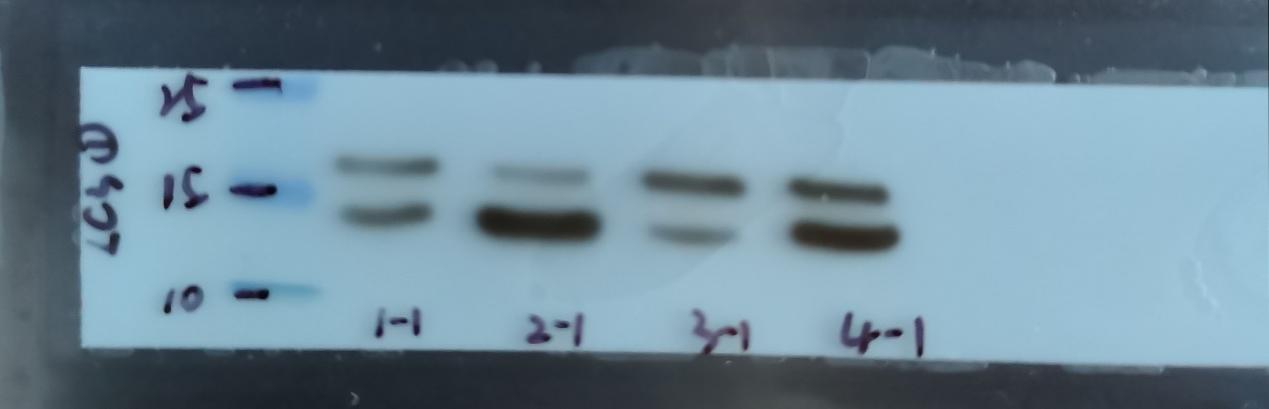


**ACTIN**


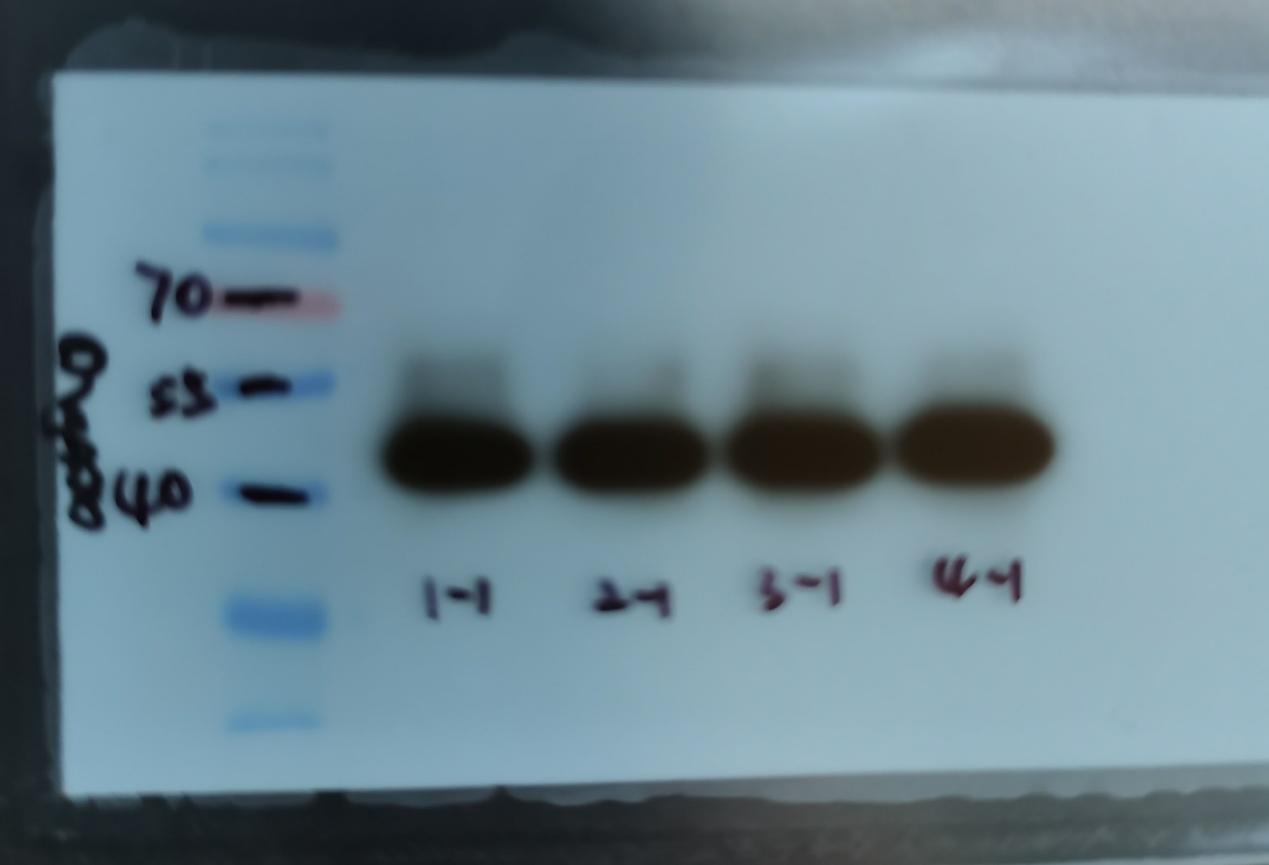


**ATP1A1**


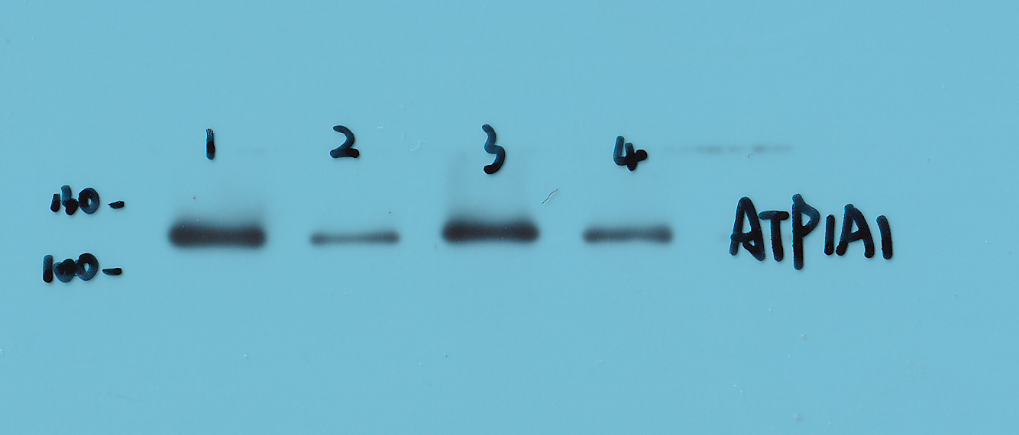


**ATP1B1**


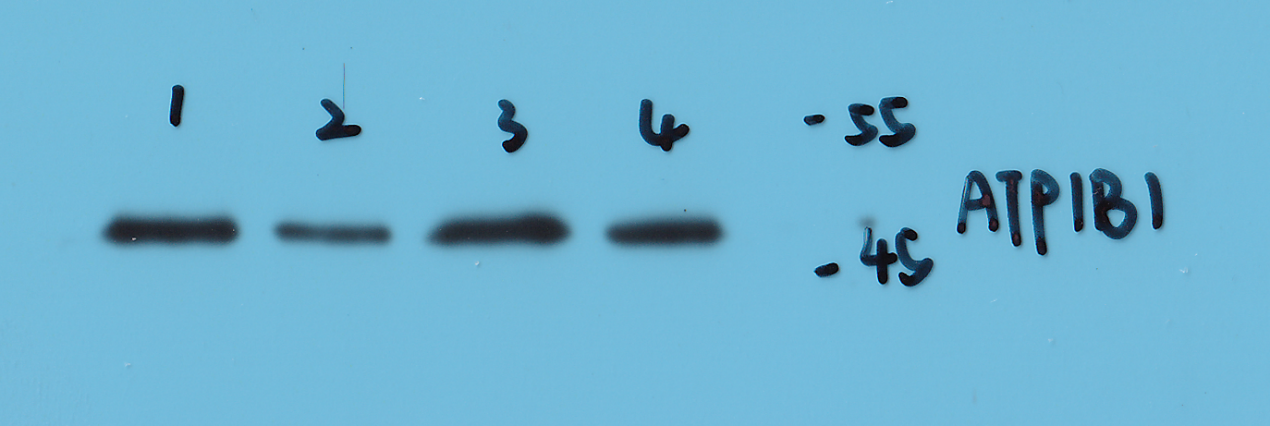


**ACTIN**


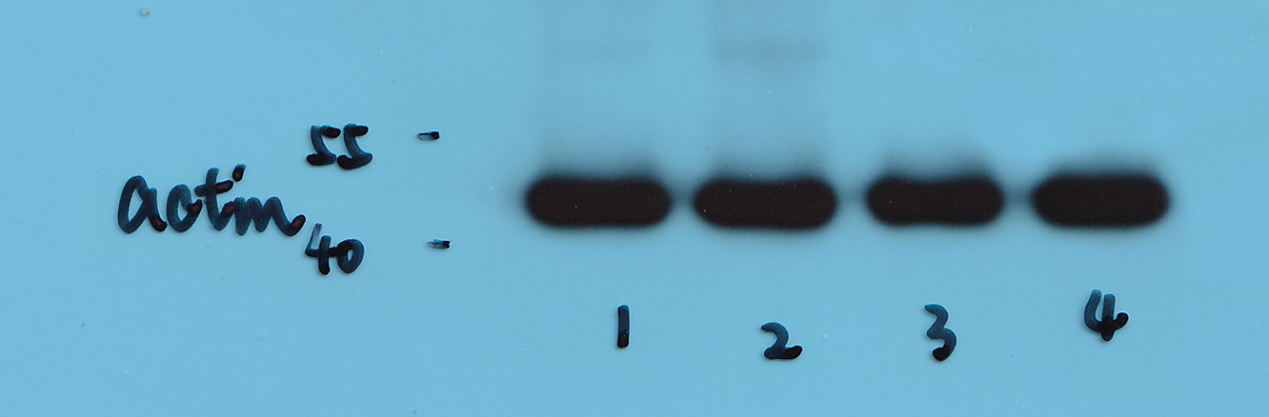


**Figure 8**. **Effects of insulin on autophagy-related proteins and ATP1A1 expression in LPS-induced ARDS mice**. Treat the mice as described in Materials and Methods. (A)(F) Representative western blotting detected the levels of LC3-II/I, Beclin-1, ATG5, P62 and ATP1A1 in lung tissues (B)(C)(D)(E)(G) Quantitative analysis of LC3-II/I, Beclin-1, ATG5, P62 and ATP1A1 were shown in bar graphs, respectively. The data are presented as mean ± SD. n = 3, the horizontal line represented the comparison between each two groups, **p*＜0.05. ***p* < 0.01. *** *p* < 0.001. **** *p* < 0.0001

LC3


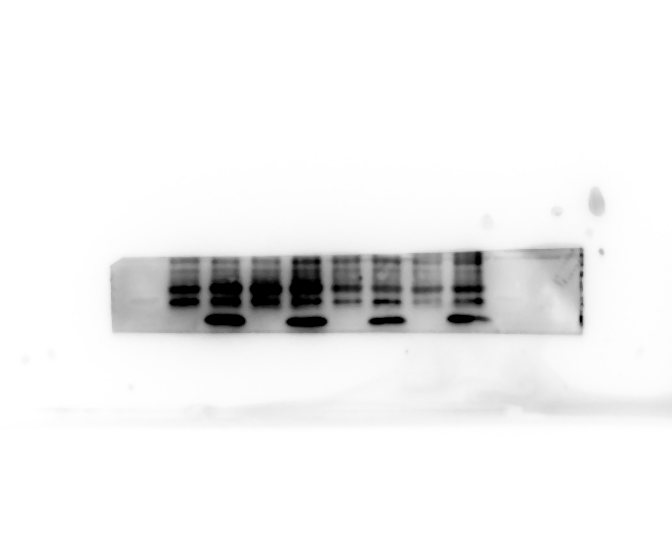


P62


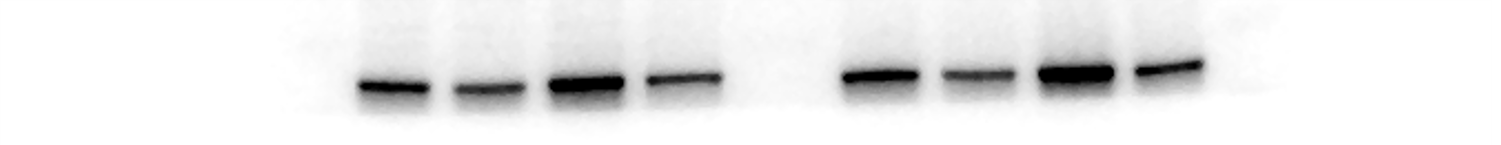


ATG5


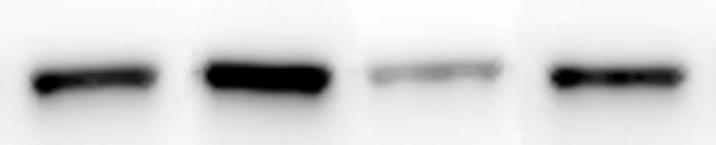


BECLIN-1


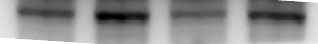


GAPDH


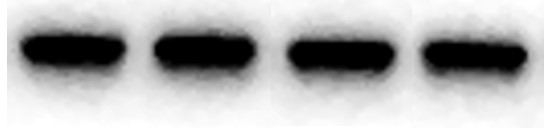


ATP1A1


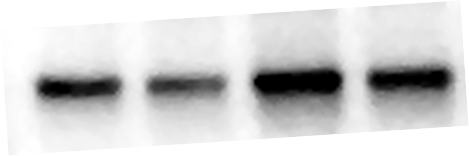


GAPDH


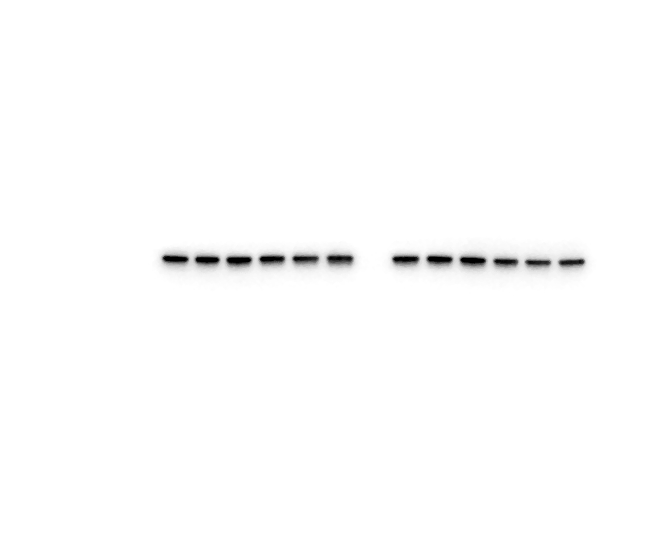


**Figure 11**. **Effect of 3-MA and Rapamycin on the role of insulin in LPS-induced autophagy in A549 cells.** (A)Western blot assay for LC3-II/I and ATP1A1. (B) (C) Quantitative analysis. The data are presented as mean ± SD. n = 3, the horizontal line represented the comparison between each two groups, **P* < 0.05, ***P* < 0.01, ****P* < 0.001

LC3


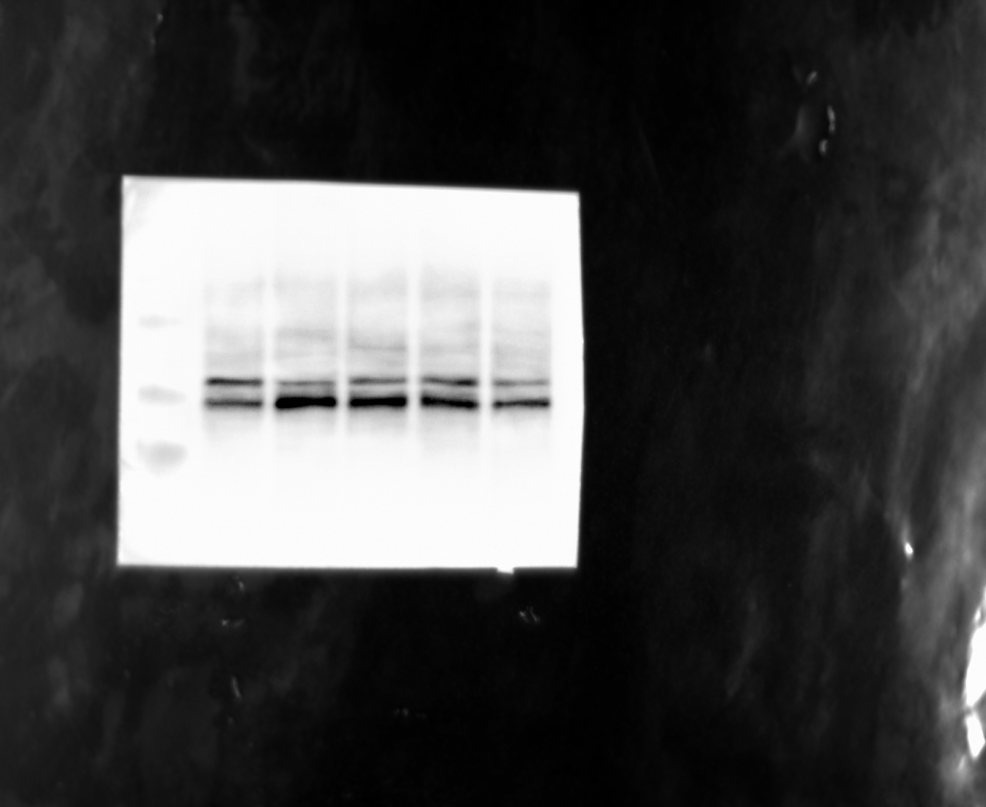


ATP1A1


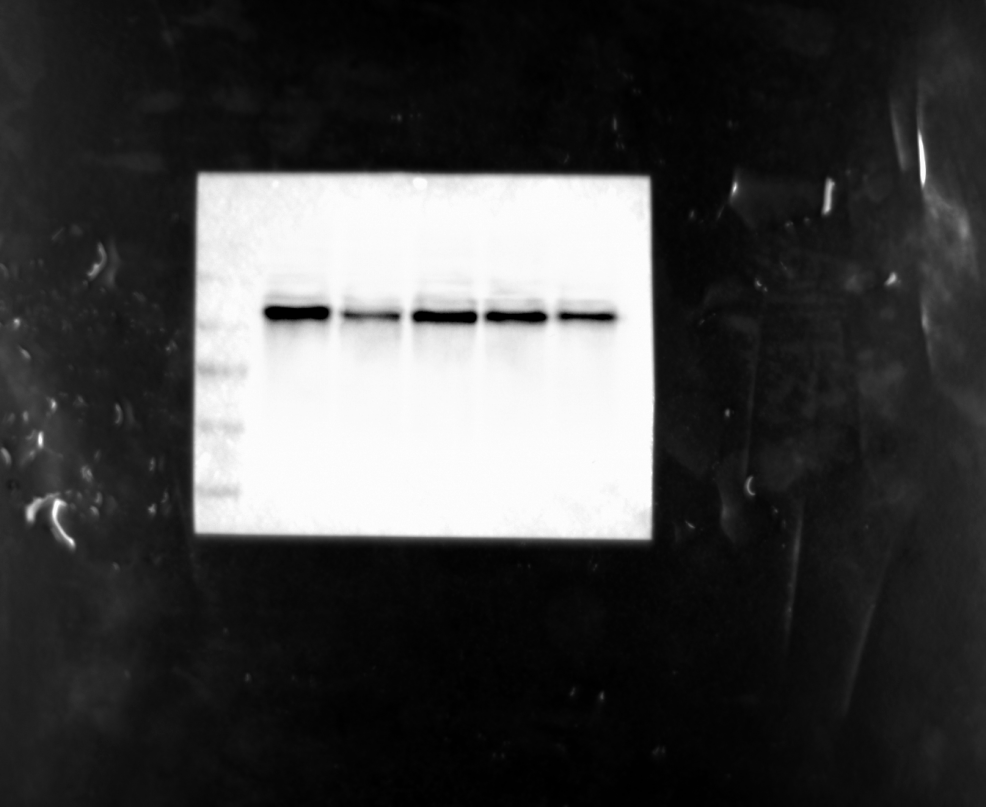


Β-ACTIN


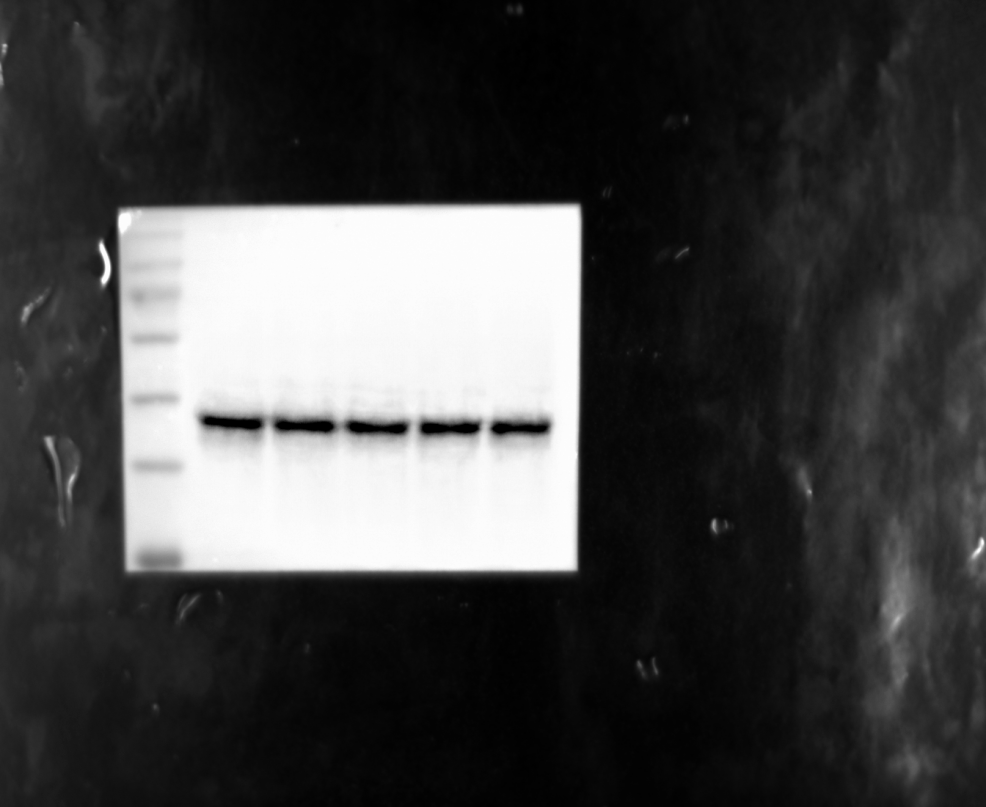


**Figure 12.** **The remarkable images of autophagosomes ultrastructure by TEM in A549 cells** (A) Representative photographs of TEM showed autophagosomes and autolysosomes. (B) Quantitative analysis of autophagosomes (orange dots) and autolysosomes (yellow dots), bars represented as mean ± SD.

**CON**


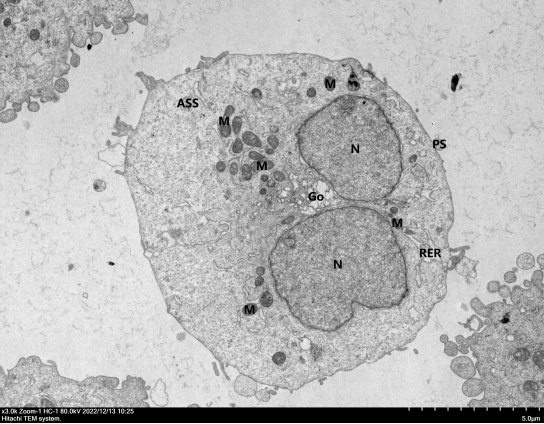


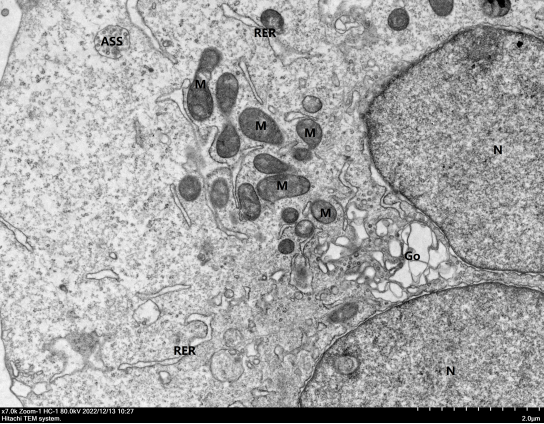


**LPS**


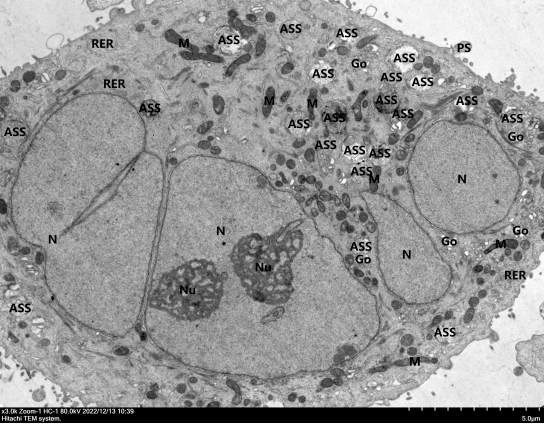


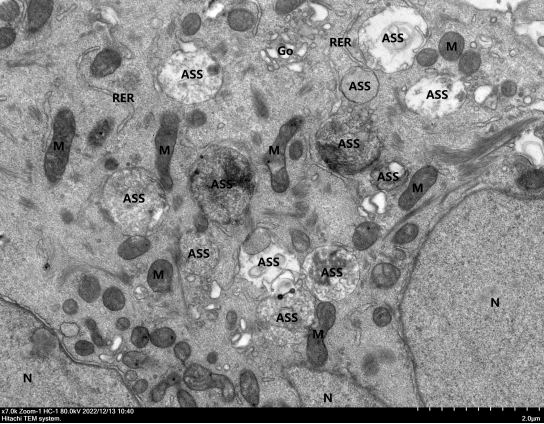


**IN**


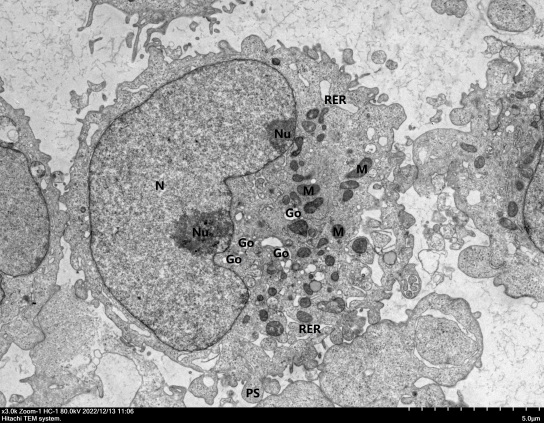


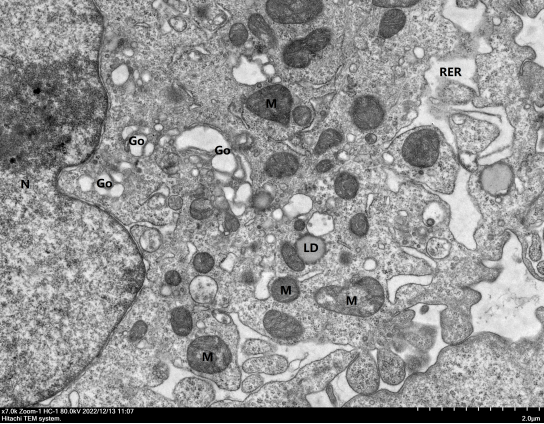


**LPS+IN**


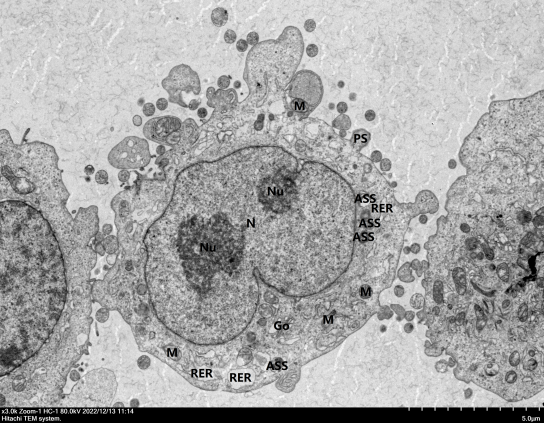

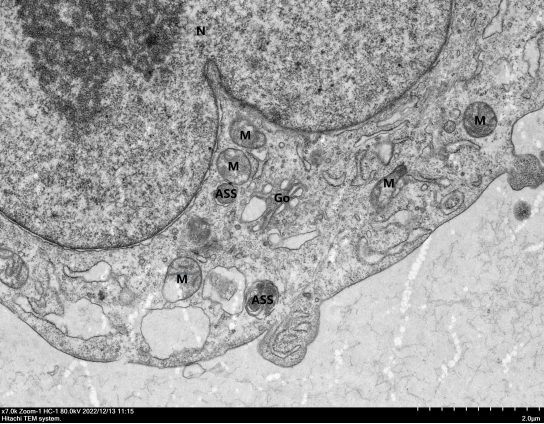

Supplement: Supplementary file 2 [file DataSheet_2.zip › The original, uncropped gels or blots.docx]
